# Targeting and repolarizing M2-like tumor-associated macrophage-mediated MR imaging and tumor immunotherapy by biomimetic nanoparticles

**DOI:** 10.1186/s12951-023-02122-8

**Published:** 2023-10-31

**Authors:** Lijuan Chong, Yao-Wen Jiang, Dongxu Wang, Pengzhao Chang, Kai Xu, Jingjing Li

**Affiliations:** 1 School of Medical Imaging, Xuzhou Medical University, Xuzhou, 221004 People’s Republic of China; 2 Department of Radiology, Affiliated Hospital of Xuzhou Medical University, Xuzhou, 221006 People’s Republic of China

**Keywords:** Biomimetic nanoparticles, Magnetic resonance imaging, Tumor-associated macrophages, Re-polarization, Immumotherapy

## Abstract

Anti-tumor M1-like and pro-tumor M2-like tumor-associated macrophages (TAMs) coexist in tumor microenvironments (TME). The adverse effects of these M1/M2 subsets on tumors directly affect the current strategies to improve anti-tumor immune response. Therefore, it has attracted great attention to change the tumor immunosuppressive microenvironment by reprogramming TAMs. In this paper, we constructed biomimetic nanoparticles (HMMDN-Met@PM) targeting M2-like TAMs for macrophage re-polarization. In detail, the core of the biomimetic nanoparticles is metformin-loaded hollow mesoporous manganese dioxide nanoparticles (HMMDN-Met). Benefited from the hollow and porous structure of HMMDN, metformin, the regulator of M1/M2 adopted in this work, can be easily and widely loaded into HMMDN. Moreover, macrophage membranes were utilized for HMMDN-Met coating (HMMDN-Met@MM) to prevent the premature drug leakage and provide specific molecular recognition/TME targeting. In addition, M2 macrophage targeting peptide (M2pep) was modified on the surface of macrophage membrane to specifically deliver the drug to M2-like TAMs to promote the polarization of M2 to M1 macrophages. Through in vitro and in vivo studies, we found that the expression of surface markers and inflammatory factors CD206, Arg-1 and IL-10 of type M2 macrophages decreased, while the surface markers of type M1 macrophages and the expression of inflammatory factors CD80, TNF-α and iNOS increased, indicating the successful re-polarization of M2 macrophages and finally realizing the inhibition of tumor growth. At the same time, under the acidic and GSH conditions of tumor, HMMDN was decomposed into Mn^2+^, which is a contrast agent for magnetic resonance imaging, thus realizing the tracking of tumor. This work practices biomimetic nanosystem in targeted imaging and immunotherapy, paving the way for strategy designing for tumor inhibition.

**Supplementary Information:**

The online version contains supplementary material available at 10.1186/s12951-023-02122-8.

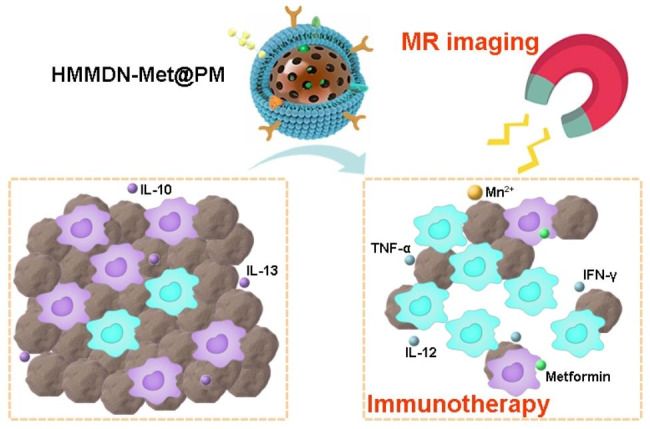

## Introduction

Cancer has become a main cause of death in China. Since 2000, the morbidity and mortality of cancer in China have been increasing [1]. Traditional chemotherapy can not only destroy rapidly dividing tumor cells, but also damage normal healthy cells, resulting in greater systemic toxicity of patients [2]. Compared with traditional chemotherapy, immunotherapy, including chimeric antigen receptor (CAR)-T cell therapy and immune checkpoint blocking, has become a more popular strategy for cancer treatment [3]. However, due to the immunosuppressive effect of tumor microenvironment (TME), these therapeutic approaches do not work, and TME can also mediate drug resistance to drug or antibody therapy [4]. Therefore, it is necessary to reshape the tumor microenvironment to restore effective anti-tumor immune response. There are many kinds of immune cells in the TME. The macrophages gathered around TME are called tumor-associated macrophages (TAMs). TAMs is a complex key regulator of TME, which can be divided into classically activated M1 macrophages and alternately activated M2 macrophages [5]. M1 macrophages kill and remove tumor cells by activating pro-inflammatory cytokines (e.g., tumor necrosis factor alpha, TNF-α) in the tumor microenvironment, recruiting or activating cytokines such as interferon (IFN)-γ and interleukin (IL)-12 to infiltrate immune effector cells into the tumor microenvironment. On the contrary, M2 macrophages release cytokines such as IL-10, IL-13, CCL9 and transforming growth factors to destroy the basement membrane, promoting angiogenesis and recruiting immunosuppressive cells to facilitate the development of primary tumor and metastasis [6] [7]. It would be a potential therapeutic strategy if pro-tumor M2 macrophages are reeducated into anti-tumor M1 macrophages, increasing the release of immunostimulatory factors and reducing the release of immunosuppressive factors to inhibit tumor growth [8]. However, due to the limitations of drug transport and macrophage targeting, the effect of this strategy is limited. Therefore, the primary task of reversing TAMs is to effectively deliver immune drugs to M2 macrophages [9].

It is known that the main pathways involved in macrophage polarization such as notch, interferon regulatory factor (IRF), Janus tyrosine kinase/signal transducer and activator of transcription (JAK/STAT), phosphatidylinositol-3-kinase (PI3K)/protein kinase B (PKB/Akt) and Toll-like receptor agonists have been used to convert pro-tumor M2 TAMs into anti-tumor M1 macrophages [10]. Resiquimod (R848) is a double agonist of Toll-like receptor TLR7/8. Lignin nanoparticles were prepared by Figueiredo et al. using lignin biopolymer as a drug carrier to be delivered into the tumor microenvironment of triple negative breast cancer model, and its tumor-like phenotype was reversed into anti-tumor M1-like macrophages [11]. A nano-gel designed by Zhang et al. was assembled from antisense signal transducer and activator of transcription 3 (anti-STAT3) siRNA. The gel can effectively block the STAT3 signal at the tumor site to prevent M2 polarization and activate the M1 polarization of TAMs [12]. Metformin (Met), as one of the most commonly used drugs for diabetes, has the characteristics of safety, low price and wide application. Studies have shown that metformin inhibits mTOR signal and activates autophagy and apoptosis to inhibit the development and metastasis of cancer through (adenosine 5’-monophosphate-activated protein kinase) AMPK-dependent and independent pathways. Furthermore, metformin has become a promising treatment choice for many diseases [13] [14]. It is reported that metformin can activate AMPK-NF-κB signal of cancer cells, regulate the expression of M1/M2, up-regulate the proportion of M1 macrophages and inhibit tumor growth and metastasis by increasing the phosphorylation of AMPK and p65 [15] [16]. The clinical application of metformin is limited because of its difficulty in transmembrane, short half-life in vivo and limited residence time in TME [17].

In recent years, it has been found that manganese dioxide nanoparticles (MDN) has good biocompatibility, adjustable structure, ability to carry drugs, and the ability to react with GSH or H_2_O_2_ to reduce Mn^4+^ to Mn^2+^. The resulting Mn^2+^ enhances the ability of T1-weighted MRI [18] [19] [20]. Most of the previously reported MDN are nano-flake and particle structure, which may not be ideal for drug loading and effective release [21] [22]. The hollow mesoporous manganese dioxide nanoparticles (HMMDN) with mesoporous shell has a good drug loading/delivery system [23]. Using HMMDN as metformin carrier can prevent premature drug leakage and improve anti-tumor efficiency. However, as an exogenous substance, nanoparticles can be recognized by the immune system as foreign bodies, which has some limitations [24]. If it is disguised as an autologous cell, it can escape the clearance of the reticuloendothelial system (RES) and prolong the blood circulation time. Using this idea, scientists are increasingly interested in nanoparticles wrapped in natural cell membranes. From then on, the complete cell membrane began to be collected from the cell and then coated on the surface of the nanoparticles [25]. The original biomimetic cell membrane nanoparticles (CMBNPs) were core-shell structures formed by co-extrusion of erythrocyte membrane shell and polylactic acid-glycolic acid (PLGA) core by top-down method [26]. Then people continue to explore different sources of cell membrane, such as cancer cells, white blood cells, and exocrine bodies, as biomimetic nano-carriers [27] [28] [29]. After completing the task of tumor homing and escaping, the membrane can fall off through morphological changes caused by extracellular microenvironment stimulation, resulting in drug release [30].

Most targeted drug delivery systems (DDS) can only target tumor cells, and the disadvantages of multidrug resistance are inevitable. There are few reports on targeting TAMs. Pun et al. reported a unique peptide sequence M2Pep (YEQDPWGVKWWY). The peptide binds to M2 TAMs and has low affinity with other leukocytes, so it becomes a specific ligand targeting M2 TAMs [31].

Thus, in this study, we used metformin as an immune stimulant, HMMDN with biocompatibility and degradability as carriers. Furthermore, the hollow mesoporous manganese dioxide nanoparticles loaded with metformin (HMMDN-Met) was used as the nano-core and co-extruded with the macrophage membrane to disguise as autologous components (HMMDN-Met@MM), and modified M2pep on the membrane surface to achieve targeted drug delivery (HMMDN-Met@PM) for M2 TAMs. The results supported that M2pep binding increased the endocytosis of nanoparticles by M2 TAMs, and metformin enhanced the polarization of M2 TAMs to M1 macrophages in vitro and in vivo, and thus increasing the anti-tumor effect after intravenous injection of HMMDN-Met@PM.

## Materials and methods

### Materials

Tetraethylorthosilicate (TEOS), Na_2_CO_3_, ethanol, methanol, ammoniumhydroxide (NH_3_·H_2_O), hexadecyl-trimethyl-ammoniumbromide (CTAB) and potassium permanganate (KMnO_4_) were purchased from SinopharmChemReagent Co., Ltd. (China). Metformin, lipopoly-saccharide (LPS) and Recombinant Murine IFN-γ were purchased from Beyotime Biotechnology Co., Ltd. (Shanghai, China). Murine IL-4 was provided by PeproTech Biotechnology Co., Ltd. (Suzhou, China). Polyclonal antibodies CD47, CD80 and CD206 were obtained from Proteintech Group, Inc. (Wuhan, China). Coumarin-6 was ordered from Aladdin Biochemical Technology Co., Ltd. (Shanghai, China). Mouse Tumor Necrosis Factor Alpha and Mouse IL-10 ELISA kit was provided by ABclonal Biotechnology Co., Ltd. (Wuhan, China). ELISA kits of Mouse iNOS and Mouse Arg-1 were obtained from Jonln Biotechnology Co., Ltd. (Shanghai, China). FITC-Anti-Mouse CD80 Antibody, FITC-Anti-Mouse CD206 and APC-Anti-Mouse CD206 Antibody were provided by Elabscience Biotechnology Co., Ltd. (Wuhan, China). β-Actin, AMPKα (D63G4) Rabbit mAb and Phospho-AMPKα (Thr172) (40H9) Rabbit mAb were ordered from Cell Signaling Technology, Inc. (MA, USA). DSPE-PEG-M2pep was purchased from SunLipo NanoTech Co., Ltd. (Shanghai, China).

### Synthesis of HMMDN

Firstly, 71.4 mL ethanol, 10 mL deionized (DI) water and 1.625 mL ammonia were mixed and heated to 30 °C. Then 3 mL TEOS was added, and the mixture was stirred quickly for 2 h to obtain silicon dioxide (SiO_2_). After centrifuged at 10,000 rpm for 10 min, and washed repeatedly with DI water and ethanol for 3 times, the product was freeze-dried for further use. Preparation of HMMDN was according to previous reference with some modifications [32] [33]. In brief, 200 mg SiO_2_ were completely dispersed in 40 mL DI water, and the mixture of 60 mL ethanol, 60 mL DI water, 4.5 mL ammonium hydroxide and 600 mg CTAB were added. After a 30-minute stirring at room temperature, 50 mg of potassium permanganate was quickly added, and the reaction continued for 6 h. After centrifugation (10,000 rpm, 10 min), washing repeatedly with ethanol and water for 3 times, the product was dispersed in 40 mL DI water. Then, 848 mg of sodium carbonate was added, and stirred at 60 °C for 10 h. After centrifugation (10,000 rpm, 10 min), washing repeatedly with DI water and ethanol for 3 times, the product was dispersed in a mixture of 20 mL methanol and 2 mL ammonium hydroxide. Next, the obtained mixture was refluxed at 60 °C for 48 h, centrifuged (10,000 rpm, 10 min), and washed repeatedly with methanol and DI water for 3 times. HMMDN was obtained and freeze-dried for next use.

### Drug loading

HMMDN (1 mg) was dispersed in 1 mL DI water, and metformin solution (2 mg/mL) was introduced. The mixture was stirred overnight at room temperature. Then the solution was centrifuged, and the supernatant was collected. The absorbance of Met in supernatant was determined at 232 nm by a UV–vis spectrometer, and the content of Met in the supernatant was calculated according to the standard curve. The drug loading capacity (LC) and entrapment efficiency (EE) were determined by the following formula.

$$\text{L}\text{C} \left( \text{w}\text{t}\text{\%} \right)=\frac{ \text{m}\text{a}\text{s}\text{s} \, \text{o}\text{f} \,\text{d}\text{r}\text{u}\text{g} \,\text{l}\text{o}\text{a}\text{d}\text{e}\text{d} \,\text{i}\text{n} \,\text{t}\text{h}\text{e} \,\text{f}\text{i}\text{n}\text{a}\text{l} \,\text{c}\text{a}\text{r}\text{r}\text{i}\text{e}\text{r}\text{s}}{\text{m}\text{a}\text{s}\text{s} \,\text{o}\text{f} \,\text{t}\text{h}\text{e} \,\text{d}\text{r}\text{u}\text{g} \,\text{l}\text{o}\text{a}\text{d}\text{e}\text{d} \,\text{f}\text{i}\text{n}\text{a}\text{l} \,\text{c}\text{a}\text{r}\text{r}\text{i}\text{e}\text{r}\text{s}}\times 100\%$$

$$\text{E}\text{E}\left( \text{w}\text{t}\text{\%} \right)=\frac{ \text{m}\text{a}\text{s}\text{s} \,\text{o}\text{f} \,\text{d}\text{r}\text{u}\text{g} \,\text{l}\text{o}\text{a}\text{d}\text{e}\text{d} \,\text{i}\text{n} \,\text{t}\text{h}\text{e} \,\text{f}\text{i}\text{n}\text{a}\text{l} \,\text{c}\text{a}\text{r}\text{r}\text{i}\text{e}\text{r}\text{s}}{\text{m}\text{a}\text{s}\text{s} \,\text{o}\text{f} \,\text{d}\text{r}\text{u}\text{g} \,\text{f}\text{e}\text{d} \,\text{i}\text{n}\text{i}\text{t}\text{i}\text{a}\text{l}\text{l}\text{y}}\times 100\%$$

### Preparation of HMMDN-Met@MM

Macrophage membrane was obtained according to the previously reported method [34] [35]. The obtained macrophage membrane was repeatedly extruded 10 times in PBS buffer through the polycarbonate porous membrane of 800 and 400 nm with an Avestin Mini-extruder, and the obtained vesicles of macrophage membrane were stored at 4 ℃.

In order to obtain HMMDN-Met@MM, the macrophage membrane vesicles and nanoparticles were mixed by ultrasonication for 2 min with the weight ratio of macrophage membrane protein to nanoparticles at 1:1. Then the mixture was extruded by an Avestin Mini-extruder for 10 times to obtain HMMDN-Met@MM solution. Finally, the uncoated membrane was removed through centrifugation (10,000 g, 30 min). The macrophage-biomimetic nanoparticles were stored at 4 °C.

### Preparation and characterization of HMMDN-Met@PM

DSPE-PEG-M2pep (0.1 mg/mL, 1 mL) and HMMDN-Met@MM (1 mg/mL) were mixed and stirred at 4 °C for 2 h following reported protocol [36]. DSPE-PEG-M2pep is amphiphilic, and can be embedded in the surface of cell membrane vesicles through its hydrophobic distearoyl (DS) carbon chain. Then the unconnected DSPE-PEG-M2pep was removed by centrifugation, and the resulting HMMDN-Met@PM was stored at 4 °C.

The morphology and size of SiO_2_, SiO_2_@mMnO_2_, HMMDN, HMMDN-Met@MM and HMMDN-Met@PM were characterized by transmission electron microscopy (TEM, JEM-1230, Japan). The structure of HMMDN nanoparticles was confirmed by HAADF-STEM imaging and element mapping of HMMDN. The specific surface area and pore size distribution of HMMDN were measured with the nitrogen adsorption/desorption isotherms calculated by Brunauer-Emmett-Teller (BET) method (ASAP 2460 3.01, Beijing, China). The size and zeta potential of SiO_2_, SiO_2_@mMnO_2_, HMMDN, HMMDN-Met@MM and HMMDN-Met@PM were measured on a Zetasizer (Nano ZS90; Malvern). To validate the successful synthesis of HMMDN-Met@MM, the UV–vis absorption spectra of Met, HMMDN, HMMDN-Met, MM vesicles and HMMDN-Met@MM were measured by UV–vis spectrometry. For the connection of DSPE-PEG-M2pep, we used Fourier transform infrared (FT-IR) spectrometer to collect the infrared spectra of HMMDN, DSPE-PEG-M2pep, HMMDN-Met@MM and HMMDN-Met@PM. Finally, dynamic light scattering was used to monitor the stability of HMMDN-Met@PM in PBS.

### In vitro drug release

The HMMDN-Met, HMMDN-Met@MM or HMMDN-Met@PM suspensions present at distinct experimental conditions (PBS (pH 7.4), PBS (pH 5.5) and PBS (pH 5.5) containing 10 mM GSH) were picked out at pre-determined time points. The amount of metformin released from the tested NPs was analyzed by UV–vis spectrometry.

### MRI ability of HMMDN-Met@PM

The aqueous T_1_-weighed magnetic resonance signal was measured on a 3.0 T MR imaging system (GE 750 W). Due to MDN can respond to tumor microenvironments (TME), such as pH or GSH [18], therefore, PBS (pH 7.4), PBS (pH 5.5) and PBS (pH 5.5) containing 10 mM GSH were adopted as the different dispersants for HMMDN-Met@PM (1.05 mM Mn). The prepared dispersion of different groups was stepwisely diluted to different concentrations of Mn^2+^ solution (0, 0.11, 0.21, 0.31, 0.42, 0.63, 1.05 mM) for further detection. After 6 h, the T_1_-weighed signal of the obtained sample was collected. The T_1_ relaxation time in the same region of interest (Regions of interest, ROI) in each hole was measured. The abscissa was the concentration of Mn, and the ordinate was the reciprocal of the T_1_ relaxation time of the sample. The slope of the corresponding linear regression equation represented the T_1_ relaxation rate.

### Cell culture

Mouse macrophages (RAW 264.7), mouse embryonic fibroblasts (3T3) and mouse breast cancer cells (4T1) were provided by the Cell Bank of the Chinese Academy of Sciences. Dulbecco’s modified Eagle medium (DMEM) containing fetal bovine serum (10%), penicillin (100 U/mL) and streptomycin (0.1 mg/mL) was used for cell culture. The cells were cultured in an incubator at 37 °C in a humidified atmosphere containing 5% CO_2_.

### Macrophage polarization

RAW 264.7 cells in culture medium were inoculated in 6-well plates (1 × 10^4^ cells/well) and cultured for 24 h. Then, the original culture medium was discarded and M1 macrophages were induced by fresh medium containing 100 ng/mL LPS and 25 ng/mL IFN-γ, and M2 macrophages were induced by 25 ng/mL IL-4. The harvested macrophages were used for the experiment of cell function in vitro.

The expression of M1 phenotypic marker CD80 and M2 phenotypic marker CD206 was detected by confocal laser scanning microscope (CLSM) to determine the macrophage phenotype. In short, after the macrophages were polarized according to the above method, the cells experienced immobilization of 4% paraformaldehyde for 15 min, followed by the treatment of 0.3% TritonX-100 for 30 min. After PBS washing, 10% bovine serum albumin solution were used to block the cells for 1 h at room temperature. Then, the cells were incubated with an anti-CD80 and CD206 antibody at 4 °C overnight. After incubated with fluorescent secondary antibody for 2 h, the anti-fluorescence quenching agent (including DAPI staining solution) were adopted for nuclei imaging. Finally, the cells were observed and photographed under confocal laser scanning microscope (CLSM).

### Cytotoxicity assessment

We evaluated the cytotoxicity of HMMDN@PM and HMMDN-Met@PM in vitro with different phenotypes of RAW 264.7 cells and 4T1 cells with MTT assay. Cells were mainly inoculated in a 96-well plate at a concentration of 1 × 10^4^/well. After 24 h of attachment, the fresh DMEM medium containing different concentrations of HMMDN-Met@PM (0, 0.02, 0.04, 0.09, 0.13, 0.17, 0.26, 0.34 mM Mn) was incubated with cells. After 24 h, 100 µL 1 mg/mL MTT solution was added to each well and incubated in the dark at 37 °C for 4 h to form formazan crystals. Then, 100 µL dimethyl sulfoxide (DMSO) was added into each well to dissolve formazan crystals. The absorbance of the dissolved crystals was recorded at 490 nm with a microplate photometer for the determination of cell viability. The cytotoxicity on RAW 264.7 cells of other nanoparticles/Met including HMMDN, Met, HMMDN-Met and HMMDN-Met@MM (Mn, 0.34 mM; Met, 30 µg/mL) were tested with similar procedures. In addition, the cytotoxicity of HMMDN-Met@PM (0, 0.02, 0.04, 0.09, 0.13, 0.17, 0.26, 0.34 mM Mn) on 3T3 cells was also measured.

### Verification of macrophage re-polarization in vitro by CLSM and flow cytometry

The expression of CD80 and CD206 was measured by CLSM to determine the macrophage re-polarization. RAW264.7 macrophages were inoculated and cultured for adhering to the wall in 6-well plates at a concentration of 1 × 10^4^/well. Then, it is induced to M2 macrophages with above-mentioned method. After that, different nano-complexes were incubated with M2 macrophages for 24 h. Finally, the cells in different groups were imaged by CLSM.

For flow cytometry test, RAW 264.7 cells were inoculated in 6-well plates at the concentration of 2 × 10^5^/well and cultured overnight. 12 h later, different macrophage phenotypes (e.g., M0, M1 and M2 types) were induced with different cytokines, and further cultured for 24 h. Then, different nanocomplexes were incubated with M2 macrophages for 24 h. Untreated M2 macrophages were served as control. Before detection, the cells were digested from 6-well plate and collected in a centrifuge tube. The supernatant was discarded by 2,000 rpm centrifugation for 5 min and cells were re-suspended in 4% paraformaldehyde solution for 10 min. After discarding the supernatant, blocked with 10% bovine serum albumin for 30 min, the obtained cells were followed by incubated with 0.3% TritonX-100 for 5 min at room temperature. Finally, 5 µL FITC anti-mouse CD80 or FITC anti-mouse CD206 was added and incubated for 1 h at 4 °C. After centrifugation, the cells were re-suspended in 500 µL 4% paraformaldehyde solution. The fluorescence expression in each sample was analyzed by flow cytometry.

### Pathway investigation of macrophage re-polarization in vitro by Western Blotting assay

To investigate the specific signaling pathways related to M1/M2 polarization, we conducted western blotting assay to evaluate the AMPK and the phosphorylation of AMPK (pAMPK) in macrophages with different treatments. Protein samples from macrophages left untreated (Control) or treated with HMMDN, HMMDN@PM, Met, HMMDN-Met, HMMDN-Met@MM or HMMDN-Met@PM (Mn, 0.34 mM; Met, 30 µg/mL) were prepared in a RIPA buffer supplemented with protease inhibitor and quantified by the BCA Protein Assay (Beyotime; China). Then, the samples were mixed with 5 × loading buffer before heating at 100 °C for 5 min. The extracted proteins ran on a 12.5% Bis-Tris 10-well minigel in running buffer using a Bio-Rad electrophoresis system at 80 V for 0.5 h and then at 100 V for 1 h. Furthermore, the proteins were transferred from the gel to the poly (vinylidene difl-fluoride) membranes followed by blocking for 1 h with 5% skimmed milk powder in tris-buffered saline after the electrophoresis. Then, the membranes were treated with primary antibodies, including anti-pAMPK, followed by the incubation of horseradish peroxidase-labeled goat/anti-rabbit IgG(H + L). The protein signals were measured by an enhanced chemiluminescent detection kit (NCM Biotech, China) using a chemilumines-cence/fluorescence image analysis system (Tanon 5200, China).

### Activation of immune response in vitro

RAW 264.7 cells were inoculated in 24-well plates (5 × 10^4^ cells/well) and cultured for 24 h followed by the induction to M2 macrophages. Then the cells were co-treated with HMMDN (0.34 mM Mn), HMMDN@PM (0.34 mM Mn), Met (30 µg/mL), HMMDN-Met (0.34 mM Mn), HMMDN-Met@MM (0.34 mM Mn) and HMMDN-Met@PM (0.34 mM Mn). After another 24 h, the content of proinflammatory cytokines including IL-10, Arg-1, iNOS and TNF-α in the supernatant were quantified with ELISA kits. All experiments were performed in triplicate.

### Specific targeting to M2 macrophages detected by MRI, CLSM and Flow cytometry

To validate the targeting effect of M2pep, RAW 264.7 cells were inoculated into 6-well plate at the density of 2 × 10^5^ cells per well, and then induced to differentiate into M1 and M2 until full growth. After incubating with HMMDN-Met, HMMDN-Met@MM or HMMDN-Met@PM for 2 h, the cells were washed with PBS. Then the cells were digested, centrifuged, re-suspended in 4% paraformaldehyde solution, and washed again. The cells were centrifuged to concentrate the cells at the bottom of the centrifuge tube. PBS-treated macrophages as blank control. Finally, the treated cells were scanned by MRI and the T_1_ signal intensity of each group was measured.

For CLSM, 1 wt% coumarin-6 (C6) was loaded into HMMDN-Met, HMMDN-Met@MM and HMDMN-Met@PM. RAW 264.7 cells were inoculated in a 6-well plate (1 × 10^4^/well), and induced into M1 and M2 after adherent. Then, after incubating for 2 h with HMMDN-Met, HMMDN-Met@MM or HMMDN-Met@PM, the cells were washed 3 times using PBS and immobilized in 4% paraformaldehyde. The nucleus was counterstained with anti-fluorescence quenching agents (including DAPI staining solution). Finally, cells were observed and photographed with CLSM. The obtained images were analyzed by ImageJ software.

For flow cytometry, the mixture of M0, M1, and M2 macrophages were adopted to mimic the in vivo macrophages in tumor microenvironment. APC-anti-mouse CD206 (red fluorescence) was used to mark M2 macrophages, while coumarin-6 (C6, green fluorescence) loaded in HMMDN-C6@PM was utilized for nanoparticle tracing. Mixed macrophages were incubated with APC-anti CD206 and/or HMMDN-C6@PM and analyzed by flow cytometry.

### Evaluation of anti-tumor effect in vitro

M2 macrophages and 4T1 cell were cultured on a Transwell culture plate with a pore diameter of 0.4 μm, establishing a non-contact co-culture model. M2 macrophages were seeded in 24-well Transwell plates (upper compartment) and cultured for 24 h. 4T1 cells were seeded in the lower compartment of each Transwell plate for 24 h. Then, different nano-complexes (HMMDN, HMMDN@PM, Met, HMMDN-Met, HMMDN-Met@MM and HMMDN-Met@PM) were incubated with M2 macrophages for 24 h, respectively. Untreated M2 macrophages were served as control. The cell viability of 4T1 was detected by MTT assay.

Due to the damage of the membrane structure of dead cells, intracellular lactate dehydrogenase (LDH) will be leaked into the culture medium. Thus, the cytotoxicity was further evaluated by measuring the release of LDH from 4T1 cells in the lower compartment. Establish a non-contact co-culture model of M2 macrophages and 4T1 cell as described above. In addition, a control model containing M2 macrophages seeded in the upper compartment, and only DMEM in the lower chamber was developed to eliminate the effect of LDH released by M2 macrophages of upper compartment. Then the supernatant was collected after incubating with different nanomaterials (HMMDN, HMMDN@PM, HMMDN-Met, Met, HMMDN-Met@MM, HMMDN-Met@PM). The supernatant was detected according to the instructions. The specific lysis of 4T1 cells was calculated by the following formula:

$$\text{s}\text{p}\text{e}\text{c}\text{i}\text{f}\text{i}\text{c} \,\text{l}\text{y}\text{s}\text{i}\text{s} \left(\text{\%}\right)=\frac{\text{O}\text{D}\left(\text{m}\text{a}\text{c}\text{r}\text{o}\text{p}\text{h}\text{a}\text{g}\text{e}\text{s} \,\text{w}\text{i}\text{t}\text{h} \,\text{d}\text{i}\text{f}\text{f}\text{e}\text{r}\text{e}\text{n}\text{t} \,\text{t}\text{r}\text{e}\text{a}\text{t}\text{m}\text{e}\text{n}\text{t}\right)-\text{O}\text{D} \left(\text{c}\text{o}\text{r}\text{r}\text{e}\text{s}\text{p}\text{o}\text{n}\text{d}\text{i}\text{n}\text{g} \,\text{c}\text{o}\text{n}\text{t}\text{r}\text{o}\text{l} \,\text{g}\text{r}\text{o}\text{u}\text{p}\right) }{\text{O}\text{D} \left(4\text{T}1 \,\text{c}\text{e}\text{l}\text{l}\text{s} \,\text{w}\text{i}\text{t}\text{h}\text{o}\text{u}\text{t} \,\text{t}\text{r}\text{e}\text{a}\text{t}\text{m}\text{e}\text{n}\text{t}\right)}\times 100 \%$$

### Animals

Female C57BL/6 mice and Balb/c mice aged 6 weeks were obtained from the Animal Center of Xuzhou Medical University. All animals were maintained under standard housing conditions and all animals were acclimatized for at least 3 days before the experiments started. All animal protocols were approved by the Ethics Committee of Xuzhou Medical University (202209S101).

### In vivo anti-tumor therapy

To construct the subcutaneous breast tumor model, 100 µL PBS containing 1 × 10^6^ 4T1 cells was subcutaneously injected into the right back of BALB/c mice. Further treatment began when the tumor volume reached approximately 75 mm^3^. Mice were randomly divided into 5 groups (n = 5 per group): PBS (control), Met, HMMDN-Met, HMMDN-Met@MM and HMMDN-Met@PM (tail intravenous injection). The Met dose is 10 mg/kg body weight (150 µL with a Met concentration of 1.0 mg/mL). The materials were given once every three days, for a total of 4 times. The weight of mice and the tumor volume were measured every two days. The volume is calculated as V = d^2^ × L/2 (d: the width of tumor, L: the length of tumor). The tumor growth inhibition (TGI) (%) = (V-V_0_)/V_0_ × 100%. On the 16th day, all tumors were separated and weighted, major organs (heart, liver, spleen, lung and kidneys) and tumors were sectioned and stained with hematoxylin and eosin (H&E), followed by observation with microscopy. In addition, TNF-α, IL-10, Arg-1 and iNOS in the sera of mice were also determined using ELISA assay.

### In vivo macrophage polarization

Immunohistochemistry staining was performed to evaluate effect of macrophages polarization of each therapy group. M1 macrophage phenotypic marker CD80 and M2 phenotypic marker CD206 were investigated by immunohistochemistry staining. The images were collected with an optical microscope.

### In vivo targeting of tumor

When the subcutaneous tumor volume grew to 75 mm^3^, the mice were anesthetized by intraperitoneal injection of 4% chloralhydrate and fixed on a special coil for magnetic resonance imaging. The whole body images of mice were performed on 3.0 T MR imaging system (GE 750 W). Before the materials injection, the plain scan images of the whole body of the mice were obtained for comparison. Subsequently, 150 µL HMMDN-Met@PM, HMMDN-Met@MM or HMMDN-Met were injected via the tail vein (the dose of Met of each group of samples was 10 mg/kg body weight). The mice were scanned by MRI at 15, 30, 45 min and 1, 2, 4, 6, 12, 24 h after injection, in which the region of interest (ROI) was tumor area and metabolic organ. The T_1_ signal intensity at each time point was measured on the AW4.6 post-processing workstation.

### In vivo safety evaluation

The in vivo toxicity of HMMDN-Met@PM nanocomplex was evaluated by monitoring the blood and histological changes. The blood samples were collected for further biochemical analysis, and histological changes of several organs (heart, liver, spleen, kidney and lung) were evaluated post-injection of HMMDN-Met@PM nanocomplex. Twelve C57BL/6 were divided into 4 groups with 3 mice in each group. Mice were injected with normal saline or HMMDN-Met@PM (10 mg/kg Met) via tail vein. At 0, 1, 7 and 21 days after administration, the blood was collected for blood routine and blood biochemical tests. The obtained organs were fixed in 10% formalin, sectioned and stained with hematoxylin and eosin (H&E) and observed under optical microscope, respectively.

As reported in the literature [37], hemolysis is caused by direct contact between nanomaterials and red blood cells, destroying red blood cells and causing hemoglobin leakage. Briefly, 1 mL of mouse blood were washed with saline for 3 times and diluted with saline. Then, 0.5 mL of the diluted whole blood sample was mixed with 0.5 mL HMMDN-Met@PM solution with different Mn concentrations. Normal saline-treated erythrocytes and DI water-treated erythrocytes were used as negative control and positive control, respectively. After incubation at 37 °C for 2 h, the solutions were centrifuged for 5 min at 3,000 rpm. The supernatant of each group was added to the 96-well plate, and the absorbance of each supernatant at 540 nm was measured by a microplate reader. Sample hemolysis rate was calculated by the following formula:

$$\text{H}\text{e}\text{m}\text{o}\text{l}\text{y}\text{s}\text{i}\text{s} \left(\text{\%}\right)=\frac{\text{O}\text{D}\text{s}\text{a}\text{m}\text{p}\text{l}\text{e} - \text{O}\text{D}\text{n}\text{e}\text{g}\text{a}\text{t}\text{i}\text{v}\text{e} \,\text{c}\text{o}\text{n}\text{t}\text{r}\text{o}\text{l}}{\text{O}\text{D}\text{p}\text{o}\text{s}\text{i}\text{t}\text{i}\text{v}\text{e} \,\text{c}\text{o}\text{n}\text{t}\text{r}\text{o}\text{l} - \text{O}\text{D}\text{n}\text{e}\text{g}\text{a}\text{t}\text{i}\text{v}\text{e} \,\text{c}\text{o}\text{n}\text{t}\text{r}\text{o}\text{l} \,\text{g}\text{r}\text{o}\text{u}\text{p}}\times 100 \%$$

### Statistical analysis

The data measured in the experiments were expressed as mean ± standard deviation. A single factor analysis of variance (ANOVA) analysis and least significant difference (LSD) were performed to compare the significant differences between the data. *p < 0.05, **p < 0.01, and ***p < 0.001 were used to indicate the significance of the difference.

## Results and discussion

### Preparation and characterization of HMMDN-Met@PM

**Fig. 1:**
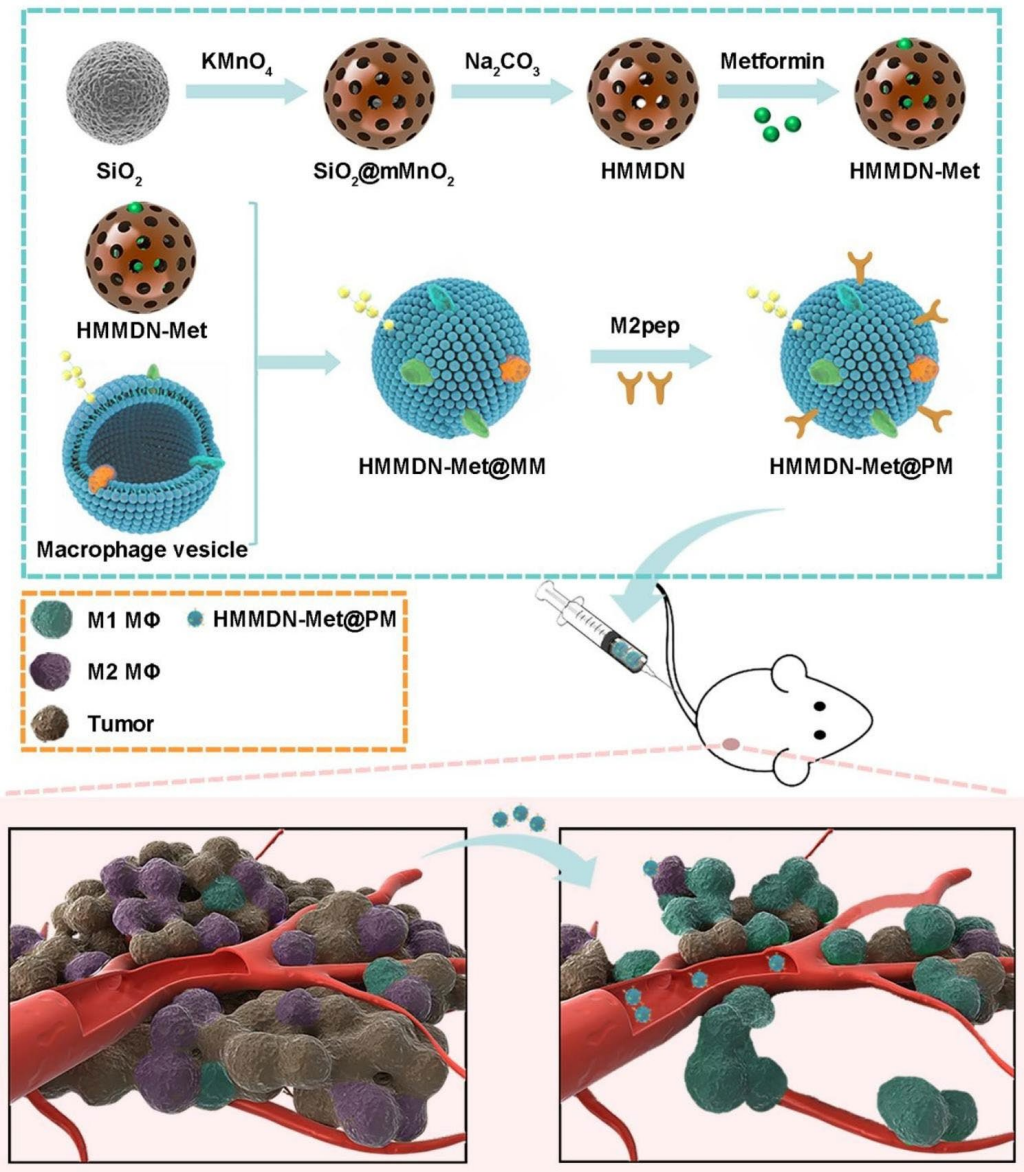
Schematic illustration of the preparation of HMMDN-Met@PM and the treatment for tumor

**Fig. 2:**
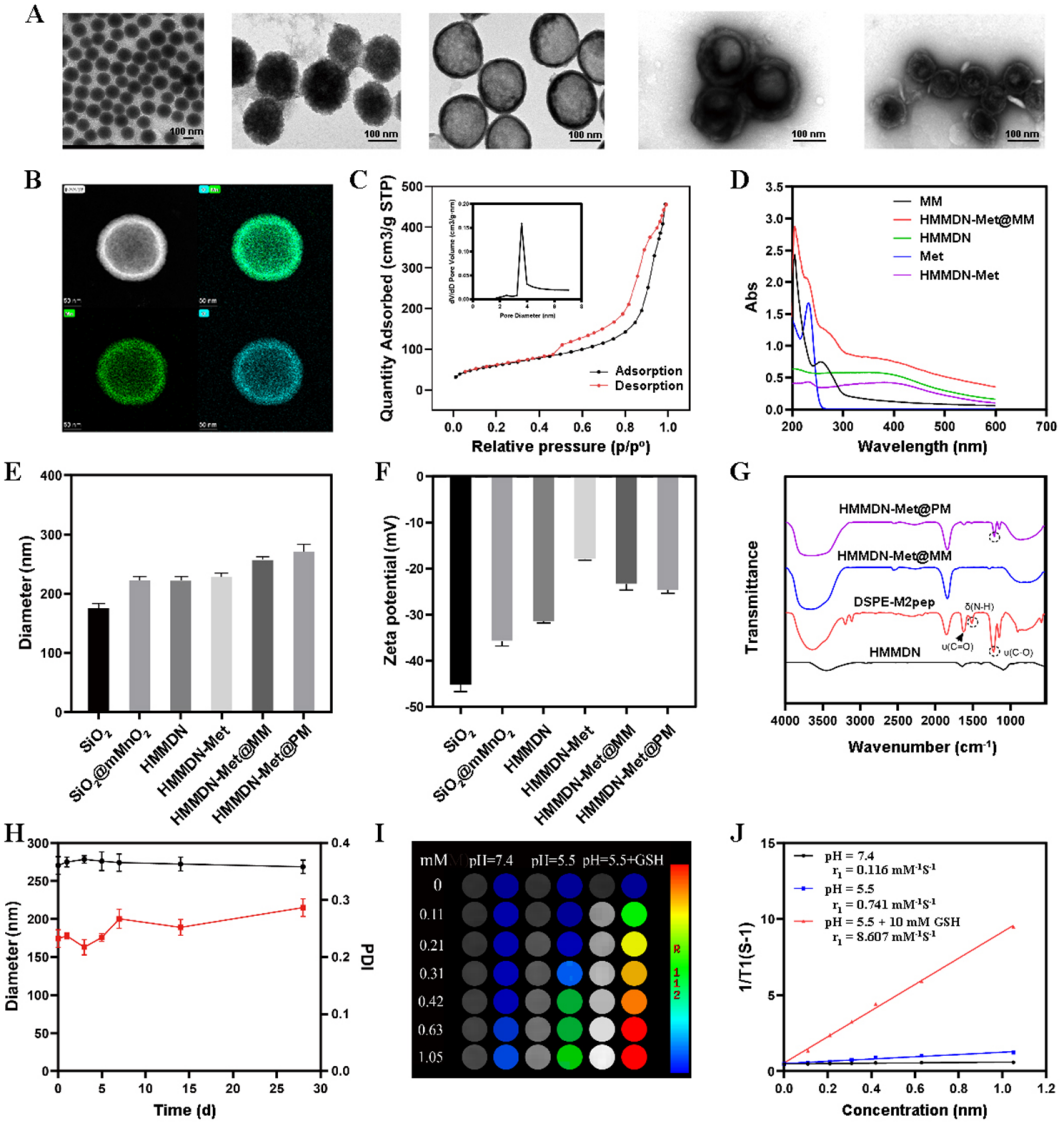
Characterizations of HMMDN-Met@PM: (**A**) TEM image of SiO_2_, SiO_2_@mMnO_2_, HMMDN, HMMDN-Met@MM and HMMDN-Met@PM. Scale bars: 100 nm. (**B**) HAADF-STEM image and elemental mapping for HMMDN. (**C**) Pore-size distribution curve and N_2_ adsorption/desorption isotherms (inset) of HMMDN. (**D**) UV–vis absorption spectra of HMMDN, Met, HMMDN-Met, MM and HMMDN-Met@MM. (**E**) Hydrodynamic diameters of SiO_2_, SiO_2_@mMnO_2_, HMMDN, HMMDN-Met, HMMDN-Met@MM and HMMDN-Met@PM. (**F**) Zeta potential of SiO_2_, SiO_2_@mMnO_2_, HMMDN, HMMDN-Met, HMMDN-Met@MM and HMMDN-Met@PM. (**G**) The FT-IR spectra of DSPE-PEG-M2Pep, HMMDN, HMMDN-Met@MM and HMMDN-Met@PM. (**H**) The size measurement of HMMDN-Met@PM NPs in PBS versus time (Black line: Diameter; Red line: polydispersity index, PDI). (**I**) T1-weighted phantom images of HMMDN-Met@PM of different concentrations of Mn at different conditions using a 3.0 T MR scanner. (**J**) T_1_ relaxivity curves of HMMDN-Met@PM

The procedure for the preparation of HMMDN-Met@PM was illustrated in Fig. 1. SiO_2_ nanoparticles were first synthesized. TEM images showed that SiO_2_ nanoparticles have a uniform spherical structure with the diameter of about 181.3 ± 2.2 nm (Fig. 2A). Then a layer of mesoporous manganese dioxide grew on the surface of SiO_2_ by in situ growth method, and the diameter increased to 228.1 ± 6.6 nm for SiO_2_@mMnO_2_. After removing the template, HMMDN with a uniform hollow structure was obtained and the diameter was determined to be about 227.4 ± 1.4 nm (Fig. 2A). The hollow structure of HMMDN was further confirmed by the high-angle annular dark-field scanning TEM (HHAADF-STEM)-based elemental mapping (Fig. 2B). To evaluate its porosity, HMMDN experienced the nitrogen adsorption-desorption isotherm. HMMDN showed a typically reversible type IV isotherm, and the surface area and pore diameter were determined to be 217 m^2^g^− 1^ and 3.5 nm (Fig. 2C), demonstrating its well-defined mesoporous structure for efficient drug loading. Then, the prepared HMMDN was employed as carrier to load Met for M2 macrophage re-polarization. UV–vis results verified Met with characteristic absorption peaks at 232 nm, which were retained well in HMMDN-Met and HMMDN-Met@MM, validating the successful loading of Met (Fig. 2D). The Met loading capacity (LC) and entrapment efficiency (EE) were determined to be 39.25% and 16.15%, respectively. The membrane coating and DSPE-PEG-M2pep modification produced a little drug loss (9.375% for HMMDN-Met@MM compared to HMMDN-Met, and 3.448% for HMMDN-Met@PM compared to HMMDN-Met@MM), which can be acceptable. Moreover, the reduced drug leakage of membrane coating under normal physiological environment and successful release under tumor environment were validated by the Met release comparison among HMMDN-Met, HMMDN-Met@MM and HMMDN-Met@PM (Fig. S1). Learnt from TEM image in Fig. 2A, compared to HMMDN, a uniform core–shell spherical nanostructure of HMMDN-Met was observed after coating of macrophage membrane onto HMMDN-Met by mechanical co-extrusion method. The hydrodynamic diameter of HMMDN-Met@MM increased from 233.9 ± 0.9 nm (HMMDN-Met) to 262.4 ± 4.5 nm (HMMDN-Met@MM) in Fig. 2E, which was ascribed to the coating of macrophage membrane with a thickness of approximately 15 nm. Additionally, the zeta potential of HMMDN-Met@MM (-24.0 ± 1.3 mV) was much more negative than that of the unmodified HMMDN-Met (-18.6 ± 0.2 mV) in Fig. 2F, further testifying the successful coating of macrophage membrane on HMMDN-Met. Next, DSPE-PEG-M2pep was incorporated into the macrophage membranes to generate M2pep modified HMMDN-Met@MM (HMMDN-Met@PM). As shown in Fig. 2G, a group of absorption peaks at ~ 1516 cm^− 1^ for N–H bending vibration and ~ 1218 cm^− 1^ for C–O stretching vibration in FT-IR spectrum demonstrated the successful insertion of DSPE-PEG-M2pep [38]. The HMMDN-Met@PM retained “core-shell” structured morphology and the average hydrodynamic diameter raised to 276.7 ± 12.8 nm (Fig. 2H). Moreover, HMMDN-Met@PM in PBS elicited good stability over a span of 28 d, which can be seen from the relatively constant hydrodynamic diameter.

### MRI ability of HMMDN-Met@PM

It is reported that Mn^2+^ is one of the most widely used MRI contrast agents for tumor diagnosis [39]. Thus, we studied MRI performance of HMMDN-Met@PM nanoparticles under different conditions. MnO_2_ nanomaterials were commonly reduced to Mn^2+^ to produce a T_1_ MRI signal in acidic condition or GSH reduction [18]. As shown in Fig. 2I, the initial longitudinal relaxivity r_1_ of HMMDN-Met at pH 7.4 or 5.5 were only 0.116 and 0.741 mM^− 1^s^− 1^ Mn, respectively. But with the introduction of 10 mM GSH at pH 5.5, an enhanced T_1_ signal was observed, and the longitudinal relaxivity r_1_ increased to 8.607 mM^− 1^s^− 1^ Mn (Fig. 2J), providing a good potential as MRI contrast agent, which was obviously higher than Gd-DTPA (4.49 mM^− 1^s^− 1^) [40]. This result demonstrated that Mn^2+^ might exhibit excellent MRI effect.

### Re-polarization of M2 macrophages by HMMDN-Met@PM in vitro

**Fig. 3:**
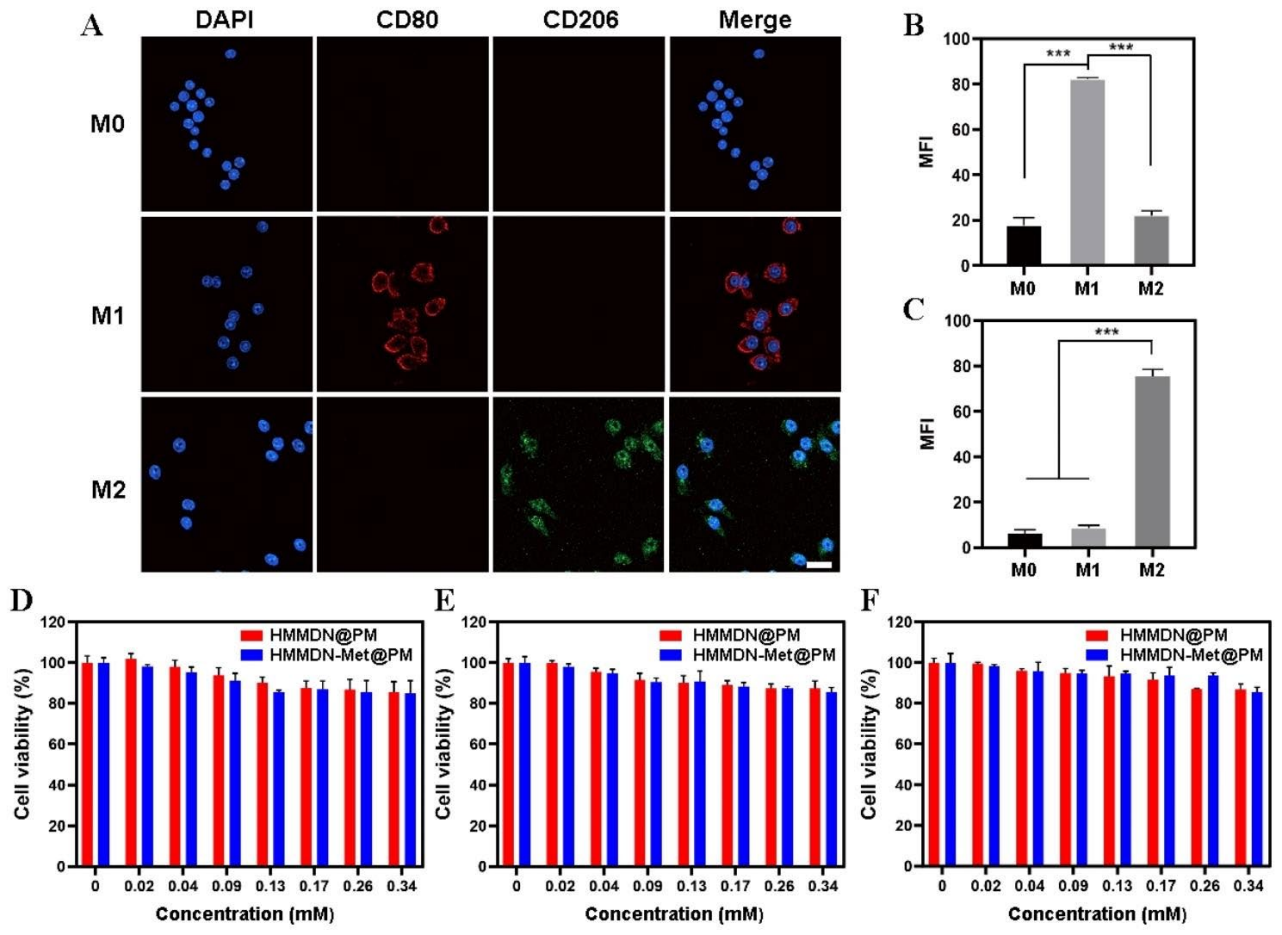
(**A**) The expression of CD80 and CD206 on various macrophage phases detected by CLSM (scale bar: 20 μm). (**B**) Quantitative analysis of CD80 fluorescence intensity on various macrophage phases (***P < 0.001). (**C**) Quantitative analysis of CD206 fluorescence intensity on various macrophage phases (***P < 0.001). (**D**) Cell viabilities of M0 macrophage treated with HMMDN@PM or HMMDN-Met@PM. (**E**) Cell viabilities of M1 macrophage treated with HMMDN@PM or HMMDN-Met@PM. (**F**) Cell viabilities of M2 macrophage treated with HMMDN@PM or HMMDN-Met@PM

Before the evaluation of HMMDN-Met@PM to re-polarize M2 macrophages, their cytotoxicity on normal healthy cells was first evaluated with MTT assay. Results in Fig. S2 showed that there was no significant cytotoxicity to 3T3 cells of HMMDN-Met@PM with Mn concentration below 0.34 mM, indicating the biosafety of HMMDN-Met@PM to normal healthy cells. According to a previous study [41], Met suppresses tumor growth through inducing TAM re-polarization. To prove the effect above, the polarization of macrophages to different phenotypes was performed and verified by detecting the expression of M1 phenotypic marker CD80 and M2 phenotypic marker CD206 by CLSM. The results in Fig. 3A–C showed that under the stimulation of LPS and IFN-γ, the red immunofluorescence intensity of CD80 increased significantly, compared with the control group and the difference was statistically significant (P < 0.001). On the contrary, under the stimulation of IL-4, the expression of CD206 increased significantly (P < 0.001). The above data indicated that M1 and M2 macrophages were polarized successfully. Additionally, HMMDN@PM, and HMMDN-Met@PM with a Mn concentration below 0.34 mM displayed no significant cytotoxicity to various macrophage phases, including M0, M1, and M2 types (Fig. 3D–F). Besides, the left nanoparticles/Met including HMMDN, Met, HMMDN-Met and HMMDN-Met@MM (Mn, 0.34 mM; Met, 30 µg/mL) were tested no cytotoxicity on M2 macrophages (Fig. S3).

**Fig. 4:**
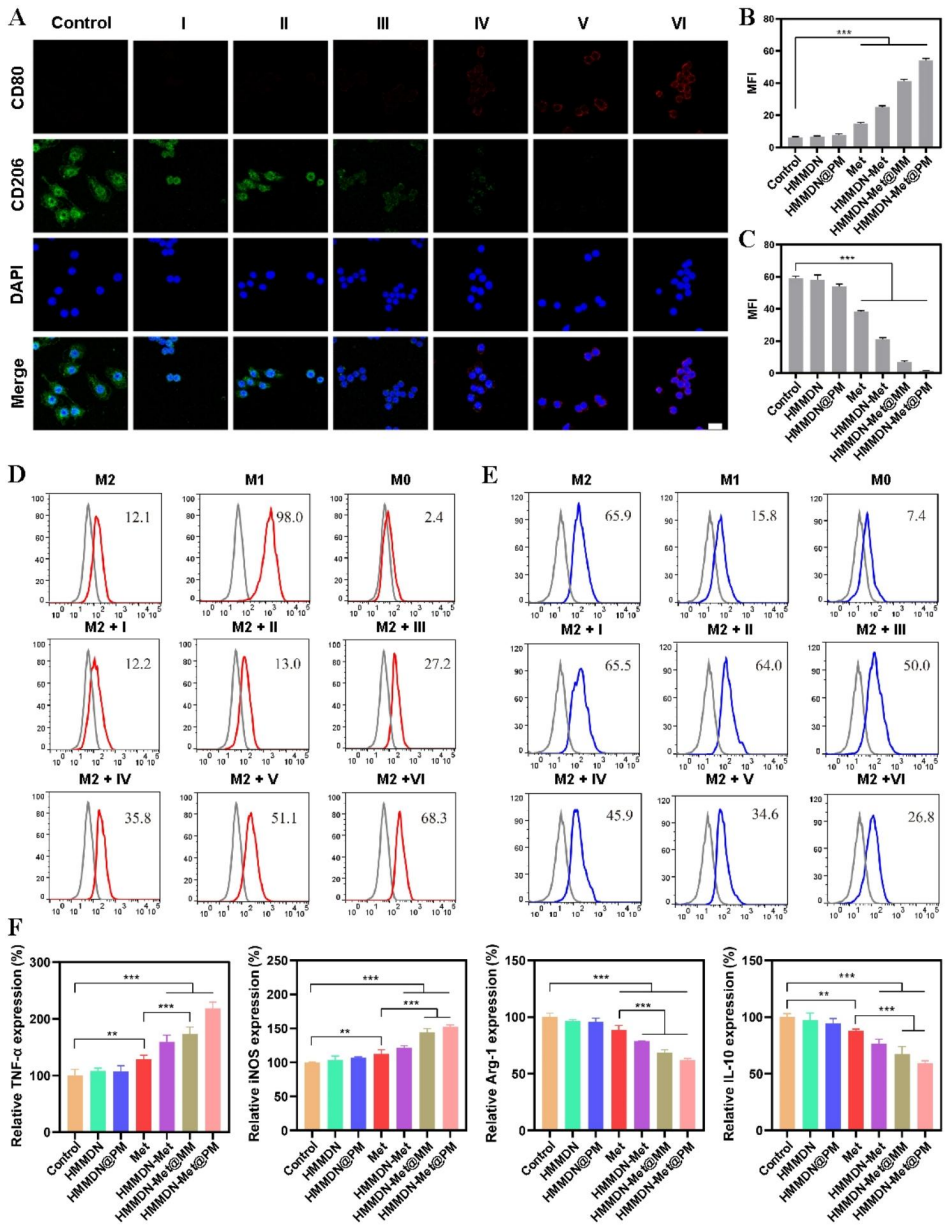
(**A**) Fluorescence images observed the expression of CD80 and CD206 in vitro after M2-like TAMs were treated with HMMDN(I), HMMDN@PM(II), Met(III), HMMDN-Met(IV), HMMDN-Met@MM(V), HMMDN-Met@PM(VI) (scale bar: 20 μm). (**B**) Quantitative analysis of CD80 fluorescence intensity on M2-like TAMs treated with different nanoparticles; (**C**) Quantitative analysis of CD206 fluorescence intensity on M2-like TAMs after different treatments (***P < 0.001). (**D**) Flow cytometric analysis of the expression of CD80 in vitro after M2-like TAMs were treated with different nanoparticles. (**E**) Flow cytometric analysis of the expression of CD206 in vitro after M2-like TAMs were treated with different nanoparticles. (**F**) The levels of immune cytokines, including TNF-α, iNOS, Arg-1 and IL-10 in M2-like TAMs supernatant after different treatments (**P < 0.01, ***P < 0.001)

Then, to confirm the re-polarization effect of HMMDN-Met@PM, M2 macrophages were hatched with HMMDN, HMMDN@PM, Met, HMMDN-Met, HMMDN-Met@MM and HMMDN-Met@PM. CLSM and flow cytometry were utilized to detect the macrophage phenotype after treatments. As expected, the red fluorescence emissions from CD80 increased obviously in HMMDN-Met, HMMDN-Met@MM and HMMDN-Met@PM group, and HMMDN-Met@PM displayed the best re-polarization effect of M2 macrophages to M1 macrophages. The green fluorescence emissions from CD206 presented similar phenomena and almost no green fluorescence emission could be observed in HMMDN-Met@PM group (Fig. 4A–C). Flow cytometry results showed that the expressions of CD80 and CD206 in untreated M2 macrophages were 13.0% and 65.9%, respectively, which were 98.0% and 15.8% in M1 macrophages. After the incubation of M2 macrophages with Met, HMMDN-Met, HMMDN-Met@MM or HMMDN-Met@PM, the expression of CD206 was dropped and CD80 was upregulated. The expressions of CD80 and CD206 in HMMDN-Met@PM treated M2 macrophages were 68.3% and 26.8%, respectively. But without Met loading, the expression of CD80 and CD206 in HMMDN or HMMDN@PM group have changed little compared to untreated M2 macrophage (Fig. 4D, E). These results confirmed an excellent re-polarization effect of HMMDN-Met@PM on M2 macrophages, benefitting from the targeting delivery of PM and re-polarization role of Met.

To further verify re-polarization effect of M2 macrophages, the changes of cytokines associated with M1 and M2 macrophages were also monitored by enzyme-linked immunosorbent assay (ELISA). Consistent with the flow cytometry results, Met, HMMDN-Met, HMMDN-Met@MM and HMMDN-Met@PM could all reduce the M2 macrophage related immunosuppressive cytokines, Arg-1 and IL-10, and improve the M1 macrophage related cytokines, iNOS and TNF-α. Compared with untreated M2 macrophages, the releases of iNOS and TNF-α increased to 152.2% and 218.7%, respectively, and the releases of Arg-1 and IL-10 decreased to 61.9% and 59.5%, respectively after treatment with HMMDN-Met@PM (Fig. 4F). Thus, it can be concluded that the M2pep modified macrophage membrane coated nanocomplex can specifically target M2 macrophages and increase the enrichment of drugs in M2 macrophages to enhance the re-polarization ability and achieve better anti-tumor effect.

Furthermore, it is reported that metformin can activate AMPK signaling pathway, and the metformin-triggered increase of AMPK phosphorylation (pAMPK) provides inhibition on the M2-like polarization induced by IL-13 [16]. Chiang et al. raised that metformin participates in regulating the expression of cytokines induced by M1 and M2 by activating the signaling pathway of AMPK/NF-β, increasing the expression of M1-related cytokines IL-12 and TNF-α, and decreasing the expression of M2-related cytokines IL-8, IL-10 and TGF-β in macrophages [15]. Therefore, we conducted Western Blotting assay to evaluate the phosphorylation of AMPK in M2 macrophages with different treatments. Results in Fig. S4A showed different expression levels of pAMPK in different groups, and the HMMDN-Met@PM group has the highest pAMPK/AMPK value compared to other groups (Fig. S4B), indicating that membrane coating (for internalization) and metformin loading (for polarization) effectively elevated the expression level of pAMPK. Therefore, Western Blotting results confirmed that HMMDN-Met@PM participated in macrophage polarization through increasing the phosphorylation of AMPK.

### Targeting efficacy of HMMDN-Met@PM in vitro

**Fig. 5:**
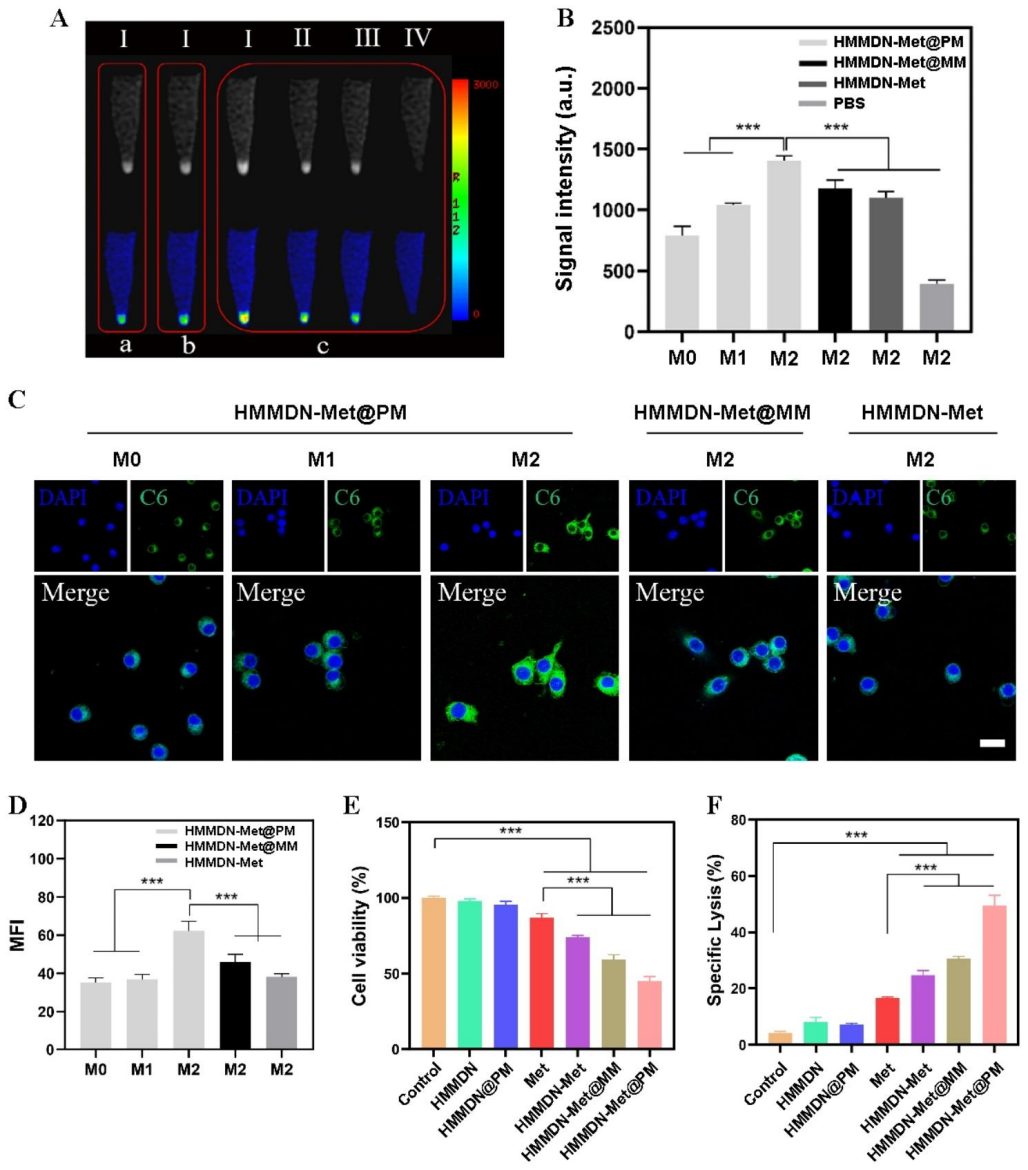
(**A**) T_1_-weighted and T_1_ pseudocolor images of different phenotypic macrophages treated with HMMDN-Met@PM(I), HMMDN-Met@MM(II), HMMDN-Met(III), PBS(IV); M0(a), M1(b) and M2(c). (**B**) Quantitative analysis of corresponding MRI signal intensity. (**C**) Fluorescence images observed the nanoparticle uptake in vitro after different phenotypic macrophages treated with different nanoparticles (scale bar: 20 μm). (**D**) Quantitative analysis of corresponding fluorescence intensity of C6. (**E**) Cell viability of 4T1 cells incubated with different nanoparticles/Met treated M2-like TAMs in transwell. (**F**) Specific lysis of 4T1 cell incubated with different nanoparticles/Met treated M2-like TAMs in transwell. (***P < 0.001)

To evaluate the targeting ability of HMMDN-Met@PM to M2-like TAMs, MRI and fluorescence imaging were performed in vitro. In Fig. 5A, the MRI signal intensities of M2 macrophages treated with HMMDN-Met, HMMDN-Met@MM or HMMDN-Met@PM were all enhanced compared with that in M2 macrophages treated with PBS and the best signal appeared in HMMDN-Met@PM group. Furthermore, when HMMDN-Met@PM were incubated with M0, M1 and M2 macrophages, respectively, the MRI signal intensity of M2 macrophages was almost 1.5-fold of M0 and M1 macrophages (Fig. 5B), indicating a higher accumulation amount of HMMDN-Met@PM in M2 macrophages with the help of the target recognition of M2pep. For fluorescence imaging, C6 was first loaded in HMMDN-Met, HMMDN-Met@MM and HMMDN-Met@PM. Similar result was obtained with that of MRI and HMMDN-Met@PM presented the strongest fluorescence emission in M2 macrophages (Fig. 5C, D). Furthermore, flow cytometric analysis of M0/M1/M2 mixture showed that the red-positive dots (CD206 tracing) and green-positive dots (nanoparticle tracing) are located in the same quadrant (Fig. S5), indicating that M2 cells are HMMDN-C6@PM-enriched cells, confirming the tendency of HMMDN-Met@PM towards M2 macrophages in vitro.

### Evaluation of anti-tumor effect in vitro

M2-like TAMs in tumor microenvironment are the main accomplices of tumor occurrence and development, and play an important role in tumor angiogenesis, metastasis and inhibition of anti-tumor immune response [42]. HMMDN-Met@PM was designed to polarize M2-like TAMs to anti-tumor M1, and increase M1 macrophage-associated immune activating factors to inhibit tumor growth. Thus, it is reasonable to first consider the effect of nanoparticles themselves on tumor cells in vitro. The viability of 4T1 cells treated with different nanoparticles directly was tested. As shown in Fig. S6, the nanoparticles themselves did not exhibit direct cytotoxicity on 4T1 cells. In addition, with the adopted concentration, there was no significant difference in the cytotoxicity of different nanoparticles, ensuring that the toxicity differences in subsequent Transwell test were caused by repolarized macrophages without the interference from the toxicity of nanoparticles themselves. Inspired by the above results, we used the Transwell co-culture model of M2 macrophages and 4T1 tumor cells to investigate its anti-tumor effect in vitro. The viability of tumor cells in lower compartment was evaluated by MTT assay. As illustrated in Fig. 5E, the cell survival rates of 4T1 cells in Met, HMMDN-Met, HMMDN-Met@MM and HMMDN-Met@PM group at the experimental concentration were 86.9%, 74.2%, 59.4%, and 45.0%, respectively, showing the best anti-tumor effect of HMMDN-Met@PM.

Due to the damage of the membrane structure of dead cells, intracellular lactate dehydrogenase (LDH) will be leaked into the culture medium. Therefore, we further collected the supernatant of 4T1 cells in the lower chamber, and evaluated the viability of 4T1 cells by LDH Kit. The results in Fig. 5F showed that LDH release rate increased significantly in the presence of Met (16.6%), HMMDN-Met (24.7%), HMMDN-Met@MM (30.7%) and HMMDN-Met@PM (49.5%) compared with control (4.2%), HMMDN (8.2%) and HMMDN@PM (7.1%). Owing to the TAMs targeting property and M1 phenotype-inducing property of HMMDN-Met@PM, 4T1 cell growth could be effectively inhibited by re-polarizing M2 macrophages.

### Specific MRI of tumor in vivo

**Fig. 6:**
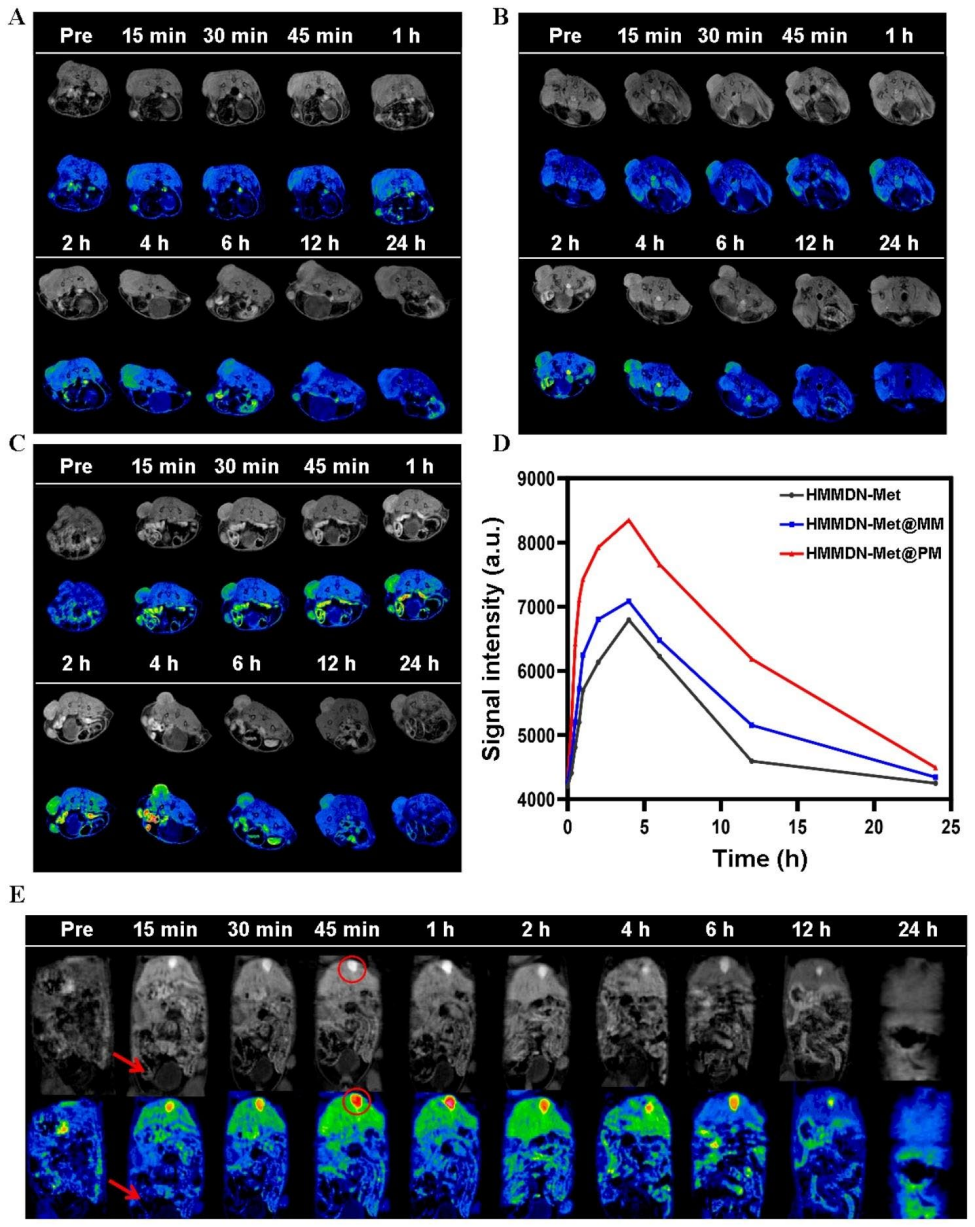
(**A**, **B** and **C**) represented T_1_-weighted and T_1_ pseudocolor images of mice (tumor) bearing breast carcinoma pre- and post-injection of HMMDN-Met, HMMDN-Met@MM and HMMDN-Met@PM at different time points, respectively. (**D**) The corresponding intensity changes of the signal in tumor. (**E**) T_1_-weighted and T_1_ pseudocolor images of mice (metabolism) bearing breast carcinoma pre- and post-injection of HMMDN-Met@PM (The red circles indicate gallbladder and the red arrows indicate bladder)

To investigate the ability of the developed nanoparticles to deliver Met to M2 macrophages in vivo, T_1_-weighted MRI scans were conducted at various time points pre- and post-injection of different nanomaterials. When the tumor volume reached about 100 mm^3^, the mice were randomly divided into 3 groups and treated with HMMDN-Met, HMMDN-Met@MM, or HMMDN-Met@PM via the tail vein injection. As shown in Fig. 6A–C, all the tumors exhibited contrast-enhanced regions post-injection of HMMDN-Met, HMMDN-Met@MM, and HMMDN-Met@PM. The T_1_ signal in tumor gradually became stronger at the first 4 h, and then turned weaker with the time. However, compared with HMMDN-Met and HMMDN-Met@MM group, T_1_ signal gradually became brighter at the first 15 min and kept stronger at the same time point in HMMDN-Met@PM group (Fig. 6D). These results showed that the coating of macrophage membrane and the M2pep modification help HMMDN-Met@PM to escape from the clearance of the immune system, reach the tumor site effectively and target tumor specifically in vivo. In addition, high MR signal in the gallbladder was observed post-injection of HMMDN-Met@PM (Fig. 6E), which was cleared completely within 24 h, implying the possible clearance route of the HMMDN-Met@PM via hepatobiliary system, which was consistent with the clearance ways of nanomaterials according to size effect [43].

### In vivo anti-tumor effect

**Fig. 7:**
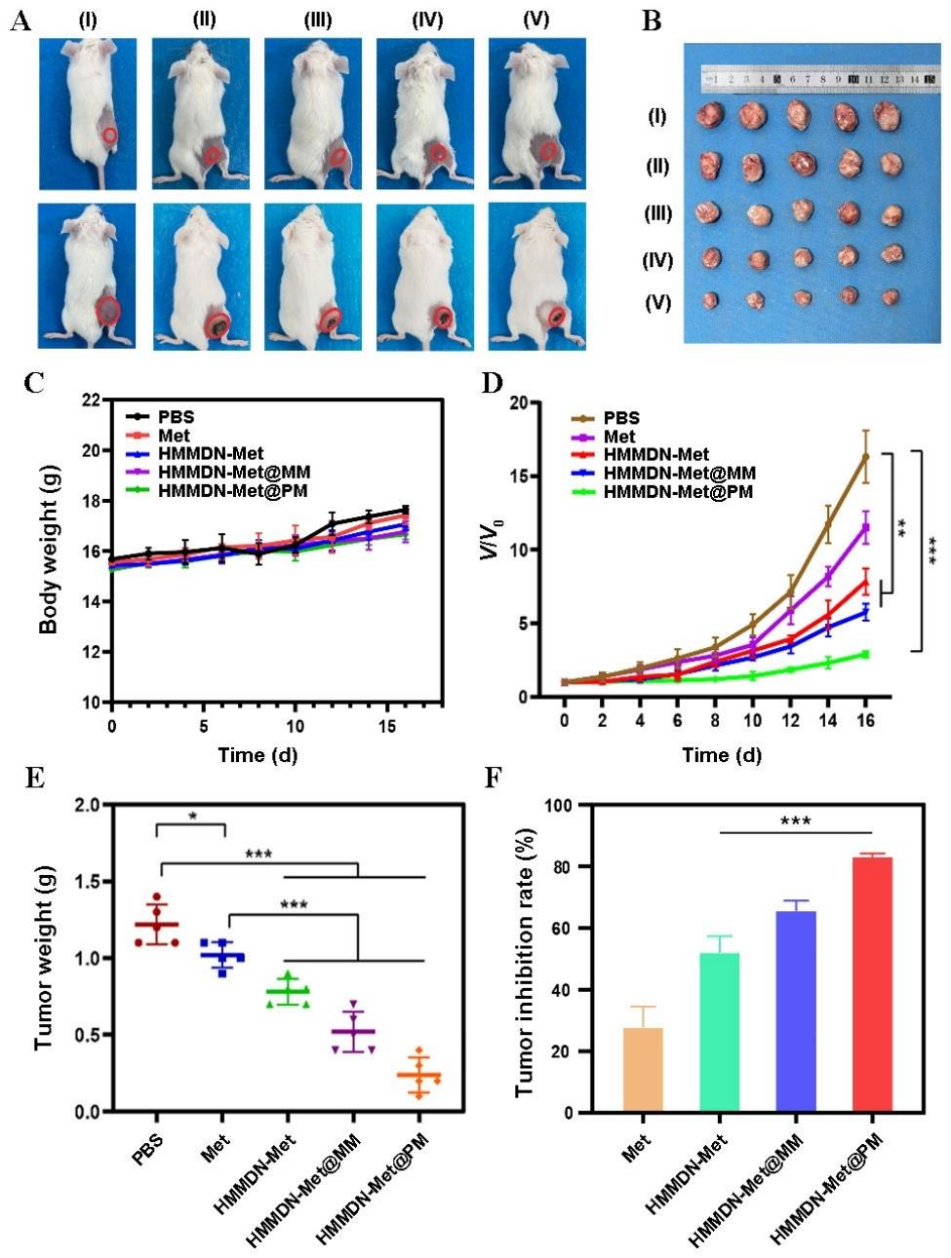
(**A**) Representative photographs of mice from different groups taken at the day 0 and day 16. (I) PBS; (II) Met; (III) HMMDN-Met; (IV) HMMDN-Met@MM; (V) HMMDN-Met@PM. (**B**) The photographs of the tumors after different treatments for 16 days. (I) PBS; (II) Met; (III) HMMDN-Met. (IV) HMMDN-Met@MM; (V) HMMDN-Met@PM. (**C**) Body weight measurement in each group. (**D**) Tumor growth curves of different groups of mice after various treatments. (**E**) Tumor weight of each group on the 16th day after treatment. (**F**) Tumor inhibition rate of each group on the 16th day. (*p < 0.05, **p < 0.01 and ***p < 0.001)

Next, the treatment effect of Balb/c mice in vivo were further studied due to the excellent therapeutic efficiency in vitro and the effective tumor targeting result in vivo. The treatment was started and recorded as day 0 when the tumor diameter reached 5 mm. The images of mice at day 0 and day 16 are displayed in Fig. 7A. Compared with other groups, the tumor hardly grew on mice after treated with HMMDN-Met@PM. Mice were sacrificed and tumor tissues were harvested from different groups on the 16th day (Fig. 7B). First, due to the increase of tumor volume and age of mice, the body weight of mice in each group increased to a certain extent during the whole monitoring time, indicating that the NPs almost have no side effect on body (Fig. 7C). In addition, the tumor volume growth curves indicated that the tumor volume increased slowest in the HMMDN-Met@PM group (Fig. 7D) and the tumor weight decreased significantly (Fig. 7E). Compared with the tumor volume at 16th day in PBS group (increased ~ 16.3-fold), the volume in free Met group increased approximately 11.5 times, exhibiting a mild anti-tumor effect with the tumor growth inhibition (TGI) of ~ 29.4%. HMMDN-Met@PM showed the best tumor inhibition effect, and the tumor volume increased about 2.9 times with a tumor inhibition rate of 84.7% (Fig. 7F). Such significant anti-tumor therapeutic effect was consistent with the fact that HMMDN-Met@PM could specifically target M2 macrophages, increase drug enrichment in M2 macrophages and promote their polarization to M1 macrophages.

**Fig. 8:**
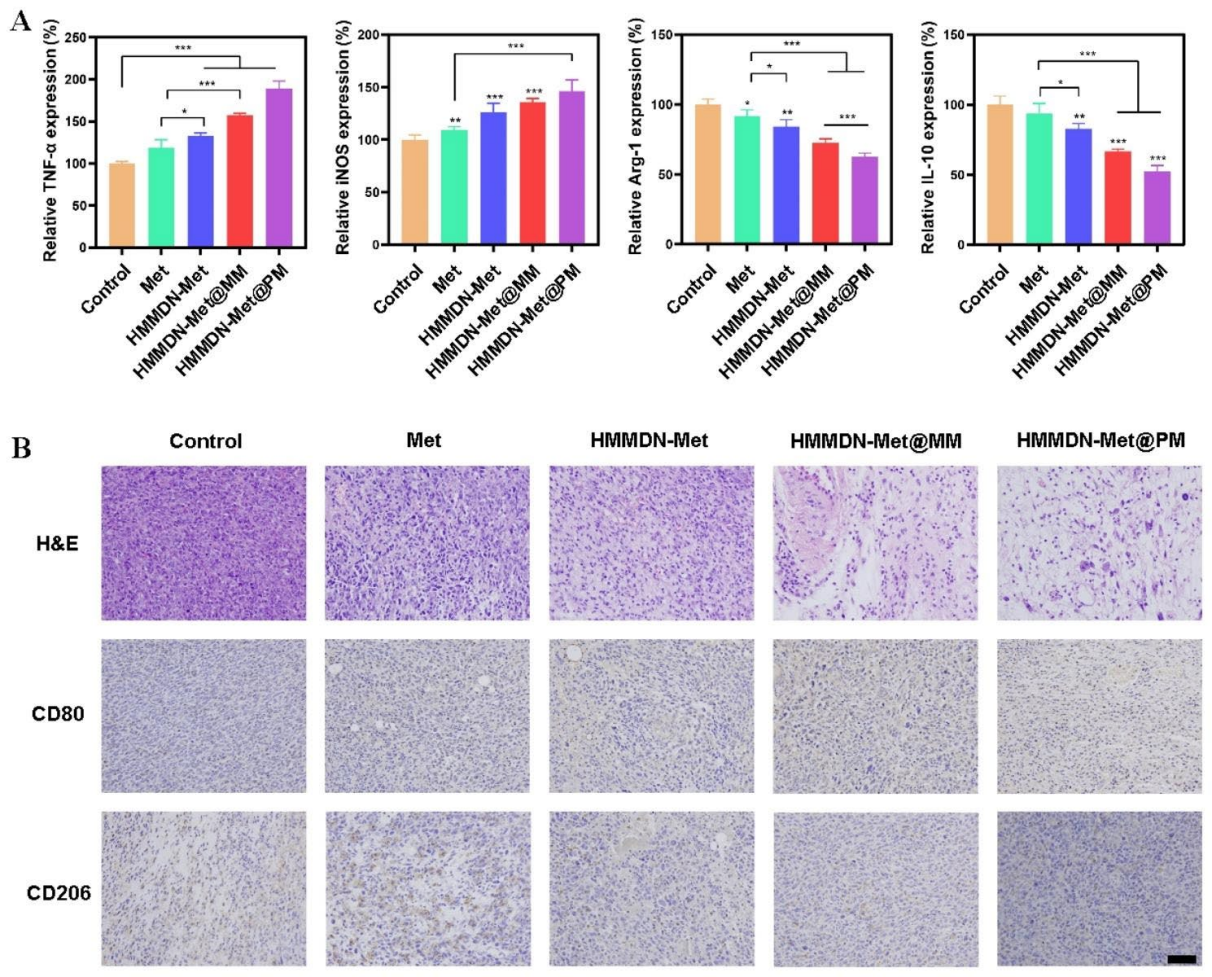
(**A**) The levels of immune cytokines TNF-α, iNOS, Arg-1 and IL-10 in the serum of mice from indicated groups. *p < 0.05, **p < 0.01 and ***p < 0.001. (**B**) H&E and immunohistochemical staining for CD80, CD206 of tumor tissues harvested from different groups (scale bar: 100 μm)

Subsequently, the typical immune cytokines secreted by M1 and M2 macrophages were determined by ELISA to further confirm the anti-tumor function of HMMDN-Met@PM. As shown in Fig. 8A, compared with PBS group, the expression of M2 macrophage related-cytokines, Arg-1 and IL-10 reduced and the expression of M1 macrophage related-cytokines, iNOS and TNF-α increased in each group (Met, HMMDN-Met, HMMDN-Met@MM and HMMDN-Met@PM). Especially, the expressions of TNF-α and iNOS increased to 189.4% and 145.1%, and the expressions of Arg-1 and IL-10 decreased to 62.9% and 52.5%, respectively in HMMDN-Met@PM group, which was consistent with anti-tumor results in vivo.

The phenotypic conversion of macrophages would effectively facilitate the apoptosis of tumor. Histochemical staining of tumor tissue was assessed to further evaluate the TAMs phenotype after different treatments in vivo. Compared with other groups, the tumor tissue in HMMDN-Met@PM group presented more positive regions of CD80 and less positive regions of CD206. Namely, HMMDN-Met@PM polarized TAMs from M2 type to M1 type successfully and had the best polarization effect among all groups (Fig. 8B). The H&E staining of tumors indicated that all the groups exhibit different degrees of cell necrosis, but the HMMDN-Met@PM group showed the best anti-tumor effect.

### Biocompatibility evaluation

**Fig. 9:**
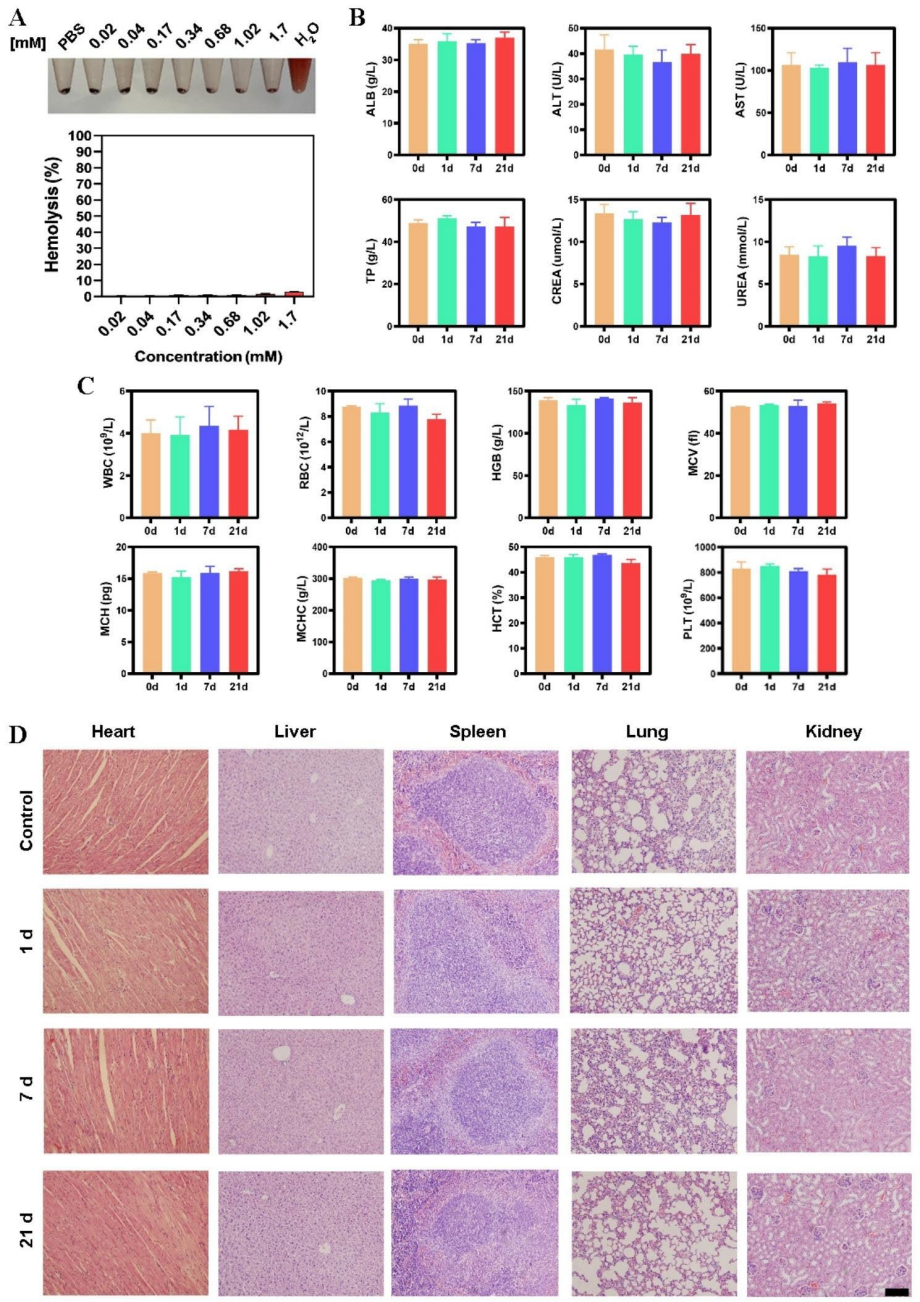
(**A**) The hemolysis of HMMDN-Met@PM of different concentrations (Negative control, normal saline; positive control, DI water). (**B, C**) blood biochemical, blood routine analysis before (0 d) and post-injection of HMMDN-Met@PM at 1, 7, 21 d. (**D**) H&E staining of separated organs including the heart, liver, spleen, lungs, kidneys of healthy mice without treatment (Control) and after injected with HMMDN-Met@PM for 1, 7 and 21 d (scale bar: 50 μm)

To further confirm the biocompatibility of HMMDN-Met@PM, hemolysis experiment was performed. As illustrated in Fig. 9A, there was no obvious hemolysis in the presence of HMMDN-Met@PM, and the hemolysis rate of each group was lower than 5%. Subsequently, blood biochemical, blood routine tests and pathological section analysis were introduced to evaluate their biocompatibility at 1, 7 and 21 days post-injection of HMMDN-Met@PM. Compared with the control group, there was no significant change in the blood routine and blood biochemical index among these groups (Fig. 9B, C). Meanwhile, H&E staining of main organs indicated that there was no obvious tissue damage in heart, liver, spleen, lung and kidneys of each group, showing the good biosafety of HMMDN-Met@PM in vivo (Fig. 9D).

## Conclusion

In summary, we designed and developed a novel HMMDN-Met@PM nanosystem for efficiently targeting TAMs in this study. Current active substances, including Toll like receptors (TLRs) agonists, transcriptional signal modulators, microRNAs, and other compounds, have been widely adopted for repolarization [44]. However, the heterogeneous distribution of TAMs in tumor tissue and the dense network of extracellular matrix limit the drug delivery efficiency in macrophage reprogramming therapy [45]. Therefore, HMMDN-Met@PM in this work provides a promising platform for TAMs targeted drug delivery to promote their reprogramming to M1 macrophages for cancer treatment. As expected, HMMDN-Met@PM showed good biosafety and reversed the macrophage phenotype, which can re-polarize M2-like TAMs to M1-like macrophages, improving the expression of pro-inflammatory cytokines and inhibiting the expression of anti-inflammatory cytokines. With the M2pep-modification, HMMDN-Met@PM can enter M2-like TAMs in vitro more than those without M2pep, inhibiting the growth of tumors effectively in vitro and in vivo. Thereby, these results suggest that the HMMDN-Met@PM offers significant potential for treating breast cancer by reversing M2-like TAMs and remodeling the tumor microenvironment, presenting a clinical application prospect. However, it should be mentioned that preclinical experiments targeting TAMs often fail to consider the complexity and multifunctionality of their interactions, resulting in ineffective treatment in clinical settings. Thus, to identify more detailed TAM characterizations and related TME molecular profiles, as well as to explore the individual roles of the components in the TME and stimulate their complex interactions would be helpful for clinical translation [46]. Furthermore, TME remodeling and stimulation of T cell-mediated immunity induced tumor antigen-specific adaptive immunity while activating immune memory for tumor antigens, possessing the potential to provide a long-term tumor prevention effect [47]. Thus, the study of prolonged immune activation or toxicity is also necessary to predict clinical translation value.

### Electronic supplementary material

Below is the link to the electronic supplementary material.

Supplementary Material 1

## Data Availability

Not applicable.

## References

[1] Xia C, Dong X, Li H, Cao M, Sun D, He S, Yang F, Yan X, Zhang S, Li N, Chen W (2022). Cancer statistics in China and United States, 2022: profiles, trends, and determinants. Chin Med J (Engl).

[2] Singh AV (2020). Commentary on peptide-conjugated nanoparticles as targeted anti-angiogenesis therapeutic and diagnostic in Cancer by Shaker A. Mousa, Pharmaceutical Research Institute, Albany College of Pharmacy and Health Sciences, Rensselaer, NY 12144, United States - peptide-conjugated nanoparticles for Multimodal Nanomedicine. Curr Med Chem.

[3] Cruz-Reyes N, Radisky DC (2023). Inflammation, infiltration, and Evasion-Tumor Promotion in the aging breast. Cancers.

[4] Klemm F, Joyce JA (2015). Microenvironmental regulation of therapeutic response in cancer. Trends Cell Biol.

[5] Shen Y, Chen JX, Li M, Xiang Z, Wu J, Wang YJ (2023). Role of tumor-associated macrophages in common digestive system malignant tumors. World J Gastrointest Oncol.

[6] Zhan C, Jin Y, Xu X, Shao J, Jin C. Antitumor therapy for breast cancer: focus on tumor-associated macrophages and nanosized drug delivery systems. Cancer Med. 2023;12: 11049–11072. 10.1002/cam4.5489 PMC10242868 36794651

[7] Wang JX, Choi SYC, Niu X, Kang N, Xue H, Killam J, Wang Y (2020). Lactic acid and an acidic Tumor Microenvironment suppress Anticancer Immunity. Int J Mol Sci.

[8] Kim H, Park HJ, Chang HW, Back JH, Lee SJ, Park YE, Kim EH, Hong Y, Kwak G, Kwon IC, Lee JE, Lee YS, Kim SY, Yang Y, Kim SH (2022). Exosome-guided direct reprogramming of tumor-associated macrophages from protumorigenic to antitumorigenic to fight cancer. Bioact Mater.

[9] Han S, Wang W, Wang S, Yang T, Zhang G, Wang D, Ju R, Lu Y, Wang H, Wang L (2021). Tumor microenvironment remodeling and tumor therapy based on M2-like tumor associated macrophage-targeting nano-complexes. Theranostics.

[10] Gao J, Liang Y, Wang L (2022). Shaping polarization of Tumor-Associated Macrophages in Cancer Immunotherapy. Front Immunol.

[11] Figueiredo P, Lepland A, Scodeller P (2021). Peptide-guided resiquimod-loaded lignin nanoparticles convert tumor-associated macrophages from M2 to M1 phenotype for enhanced chemotherapy. Acta Biomater.

[12] Zhang F, Parayath NN, Ene CI (2019). Genetic programming of macrophages to perform anti-tumor functions using targeted mRNA nanocarriers. Nat Commun.

[13] Hasanvand A (2022). The role of AMPK-dependent pathways in cellular and molecular mechanisms of metformin: a new perspective for treatment and prevention of diseases. Inflammopharmacology.

[14] Cejuela M, Martin-Castillo B, Menendez JA, Pernas S (2022). Metformin and breast Cancer: where are we now?. Int J Mol Sci.

[15] Chiang CF, Chao TT, Su YF, Hsu CC, Chien CY, Chiu KC, Shiah SG, Lee CH, Liu SY, Shieh YS (2017). Metformin-treated cancer cells modulate macrophage polarization through AMPK-NF-κB signaling. Oncotarget.

[16] Ding L, Liang G, Yao Z, Zhang J, Liu R, Chen H, Zhou Y, Wu H, Yang B, He Q (2015). Metformin prevents cancer metastasis by inhibiting M2-like polarization of tumor associated macrophages. Oncotarget.

[17] Xiong W, Qi L, Jiang N, Zhao Q, Chen L, Jiang X, Li Y, Zhou Z, Shen J (2021). Metformin liposome-mediated PD-L1 downregulation for amplifying the photodynamic immunotherapy efficacy. ACS Appl Mater Interfaces.

[18] Luo M, Lv Y, Luo X, Ren Q, Sun Z, Li T, Wang A, Liu Y, Yang C, Li X (2022). Developing Smart Nanoparticles Responsive to the Tumor Micro-Environment for enhanced synergism of Thermo-Chemotherapy with PA/MR Bimodal Imaging. Front Bioeng Biotechnol.

[19] Cai X, Zhu Q, Zeng Y, Zeng Q, Chen X, Zhan Y (2019). Manganese oxide nanoparticles as MRI contrast agents in Tumor Multimodal Imaging and Therapy. Int J Nanomedicine.

[20] Lopes SV, Walczak P, Janowski M, Reis RL, Silva-Correia J, Oliveira JM (2022). Cytocompatible manganese dioxide-based hydrogel nanoreactors for MRI imaging. Biomater Adv.

[21] Prasad P, Gordijo CR, Abbasi AZ, Maeda A, Ip A, Rauth AM, DaCosta RS, Wu XY (2014). Multifunctional albumin-MnO_2_ nanoparticles modulate solid tumor microenvironment by attenuating hypoxia, acidosis, vascular endothelial growth factor and enhance radiation response. ACS Nano.

[22] Abbasi AZ, Prasad P, Cai P, He C, Foltz WD, Amini MA, Gordijo CR, Rauth AM, Wu XY (2015). Manganese oxide and docetaxel co-loaded fluorescent polymer nanoparticles for dual modal imaging and chemotherapy of breast cancer. J Control Release.

[23] Yang G, Xu L, Chao Y, Xu J, Sun X, Wu Y, Peng R, Liu Z (2017). Hollow HMDN as a tumor-microenvironment-responsive biodegradable nano-platform for combination therapy favoring antitumor immune responses. Nat Commun.

[24] Vijayan V, Uthaman S, Park IK (2018). Cell membrane coated nanoparticles: an emerging Biomimetic Nanoplatform for targeted bioimaging and therapy. Adv Exp Med Biol.

[25] Luk BT, Zhang L (2015). Cell membrane-camouflaged nanoparticles for drug delivery. J Control Release.

[26] Hu CM, Zhang L, Aryal S, Cheung C, Fang RH, Zhang L (2011). Erythrocyte membrane-camouflaged polymeric nanoparticles as a biomimetic delivery platform. Proc Natl Acad Sci U S A.

[27] Li X, Yu Y, Chen Q, Lin J, Zhu X, Liu X, He L, Chen T, He W (2022). Engineering cancer cell membrane-camouflaged metal complex for efficient targeting therapy of breast cancer. J Nanobiotechnol.

[28] Ma W, Zhu D, Li J, Chen X, Xie W, Jiang X, Wu L, Wang G, Xiao Y, Liu Z, Wang F, Li A, Shao D, Dong W, Liu W, Yuan Y (2020). Coating biomimetic nanoparticles with chimeric antigen receptor T cell-membrane provides high specificity for hepatocellular carcinoma photothermal therapy treatment. Theranostics.

[29] Mo J, Da X, Li Q, Huang J, Lu L, Lu H (2022). The study of Exosomes-Encapsulated mPEG-PLGA polymer drug-loaded particles for targeted therapy of Liver Cancer. J Oncol.

[30] Zhang Y, Cai K, Li C, Guo Q, Chen Q, He X, Liu L, Zhang Y, Lu Y, Chen X, Sun T, Huang Y, Cheng J, Jiang C (2018). Macrophage-membrane-coated nanoparticles for Tumor-Targeted chemotherapy. Nano Lett.

[31] Cieslewicz M, Tang J, Yu JL, Cao H, Zavaljevski M, Motoyama K, Lieber A, Raines EW, Pun SH (2013). Targeted delivery of proapoptotic peptides to tumor-associated macrophages improves survival. Proc Natl Acad Sci U S A.

[32] Xu X, Duan J, Liu Y, Kuang Y, Duan J, Liao T, Xu Z, Jiang B, Li C (2021). Multi-stimuli responsive hollow HMDN-based drug delivery system for magnetic resonance imaging and combined chemo-chemodynamic cancer therapy. Acta Biomater.

[33] He H, Yang Q, Li H, Meng S, Xu Z, Chen X, Sun Z, Jiang B, Li C (2021). Hollow mesoporous HMDN-carbon nanodot-based nanoplatform for GSH depletion enhanced chemodynamic therapy, chemotherapy, and normal/cancer cell differentiation. Mikrochim Acta.

[34] Sha X, Dai Y, Chong L, Wei M, Xing M, Zhang C, Li J (2022). Pro-efferocytic macrophage membrane biomimetic nanoparticles for the synergistic treatment of atherosclerosis via competition effect. J Nanobiotechnol.

[35] Xuan M, Shao J, Dai L, He Q, Li J (2015). Macrophage cell membrane camouflaged mesoporous silica nanocapsules for in vivo Cancer therapy. Adv Healthc Mater.

[36] Zhang Y, Chen Y, Li J, Zhu X, Liu Y, Wang X, Wang H, Yao Y, Gao Y, Chen Z (2021). Development of toll-like receptor agonist-loaded nanoparticles as Precision Immunotherapy for Reprogramming Tumor-Associated Macrophages. ACS Appl Mater Interfaces.

[37] Luo L, Wu W, Sun D, Dai HB, Wang Y, Zhong Y (2018). Acid-activated melittin for targeted and safe antitumor therapy. Bioconjug Chem.

[38] Liu FR, Wang L, Wang R (2013). Calcium-binding capacity of wheat germ protein hydrolysate and characterization of peptide-calcium complex. J Agric Food Chem.

[39] Gao F, Tang Y, Liu WL, Zou MZ, Huang C, Liu CJ, Zhang XZ (2019). Intra/Extracellular lactic acid exhaustion for synergistic metabolic therapy and immunotherapy of tumors. Adv Mater.

[40] Zheng S, Yu N, Han C, Xie T, Dou B, Kong Y, Zuo F, Shi M, Xu K (2019). Preparation of gadolinium doped carbon dots for enhanced MR imaging and cell fluorescence labeling. Biochem Biophys Res Commun.

[41] Wei Z, Zhang X, Yong T, Bie N, Zhan G, Li X, Liang Q, Li J, Yu J, Huang G, Yan Y, Zhang Z, Zhang B, Gan L, Huang B, Yang X (2021). Boosting anti-PD-1 therapy with metformin-loaded macrophage-derived microparticles. Nat Commun.

[42] Liu L, Wang Y, Guo X, Zhao J, Zhou S (2020). A biomimetic polymer magnetic Nanocarrier Polarizing Tumor-Associated Macrophages for Potentiating Immunotherapy. Small.

[43] Chithrani BD, Chan WC (2007). Elucidating the mechanism of cellular uptake and removal of protein-coated gold nanoparticles of different sizes and shapes. Nano Lett.

[44] Cassetta L, Pollard JW (2018). Targeting macrophages: therapeutic approaches in cancer. Nat Rev Drug Discovery.

[45] Xu X, Gong X, Wang Y et al. Reprogramming tumor associated macrophages toward M1 phenotypes with nanomedicine for anticancer immunotherapy. Adv Therap. 2020, 1900181.

[46] Joyce JA, Pollard JW (2009). Microenvironmental regulation of metastasis. Nat Rev Cancer.

[47] Feng Q, Ma X, Cheng K (2022). Engineered bacterial outer membrane vesicles as controllable two-way adaptors to activate macrophage phagocytosis for Improved Tumor Immunotherapy. Adv Mater.

