# Supplementary material for: Targeting and repolarizing M2-like tumor-associated macrophage-mediated MR imaging and tumor immunotherapy by biomimetic nanoparticles
Source: J Nanobiotechnology. 2023 Oct 31;21:401. doi: 10.1186/s12951-023-02122-8 (PMC10617215; doi:10.1186/s12951-023-02122-8)
Supplement: Supplementary file 1 — Supplementary Material 1 [file 12951_2023_2122_MOESM1_ESM.docx]

**Supplementary material for**

**T****argeting and repolarizing M2-like tumor-associated macrophage-mediated MR imaging and tumor immunotherapy by biomimetic nanoparticles**

**Lijuan Chong^1,2,#^, Yao-Wen Jiang^1,2,#^, Dongxu Wang^1^, Pengzhao Chang^1^, Kai Xu^1,2,*^, Jingjing Li^1,2,*^**

^1^School of Medical Imaging, Xuzhou Medical University, Xuzhou, 221004, People’s Republic of China;

^2^Department of Radiology, Affiliated Hospital of Xuzhou Medical University, Xuzhou, 221006, People’s Republic of China
^#^These authors contributed equally to this work
*Correspondence: Kai Xu; Jingjing Li, Email: xkpaper@163.com; qingchao0124@163.com


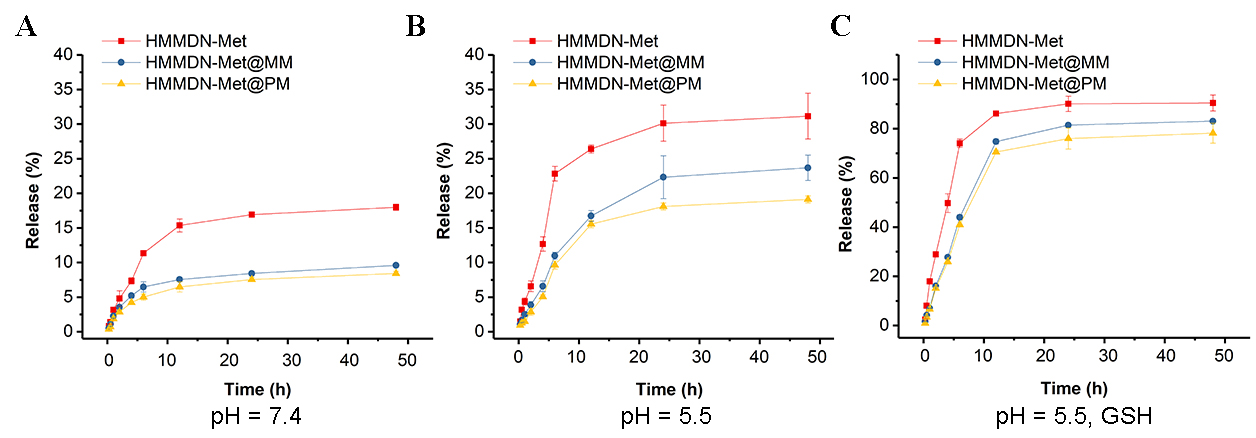


**Figure S1.** Metformin released from HMMDN-Met, HMMDN-Met@MM or HMMDN-Met@PM under **A** pH = 7.4, **B** pH = 5.5 and **C** pH = 5.5, GSH.


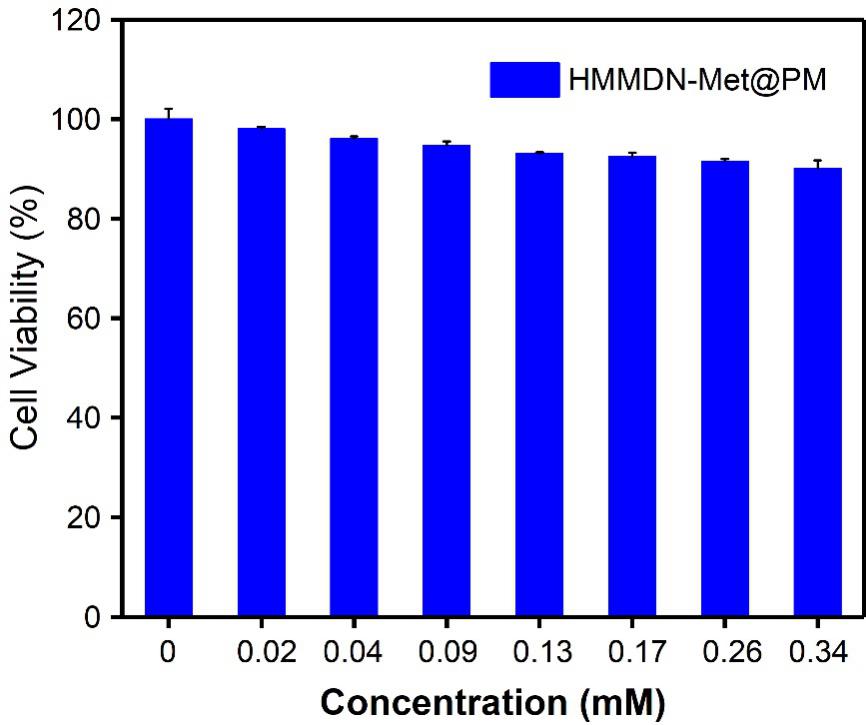


**Figure S2.** Cell viability of 3T3 cells treated with HMMDN-Met@PM for 24 h.


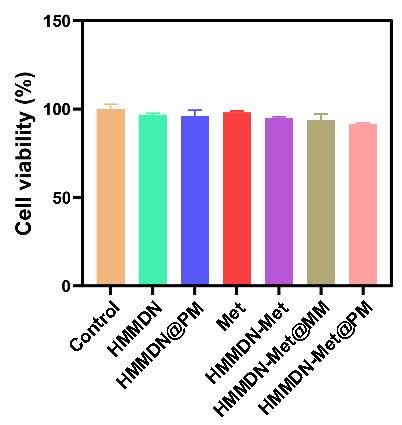


**Figure S3.** Cell viability of macrophages incubated with different nanoparticles/Met (Mn, 0.34 mM; Met, 30 μg/mL).


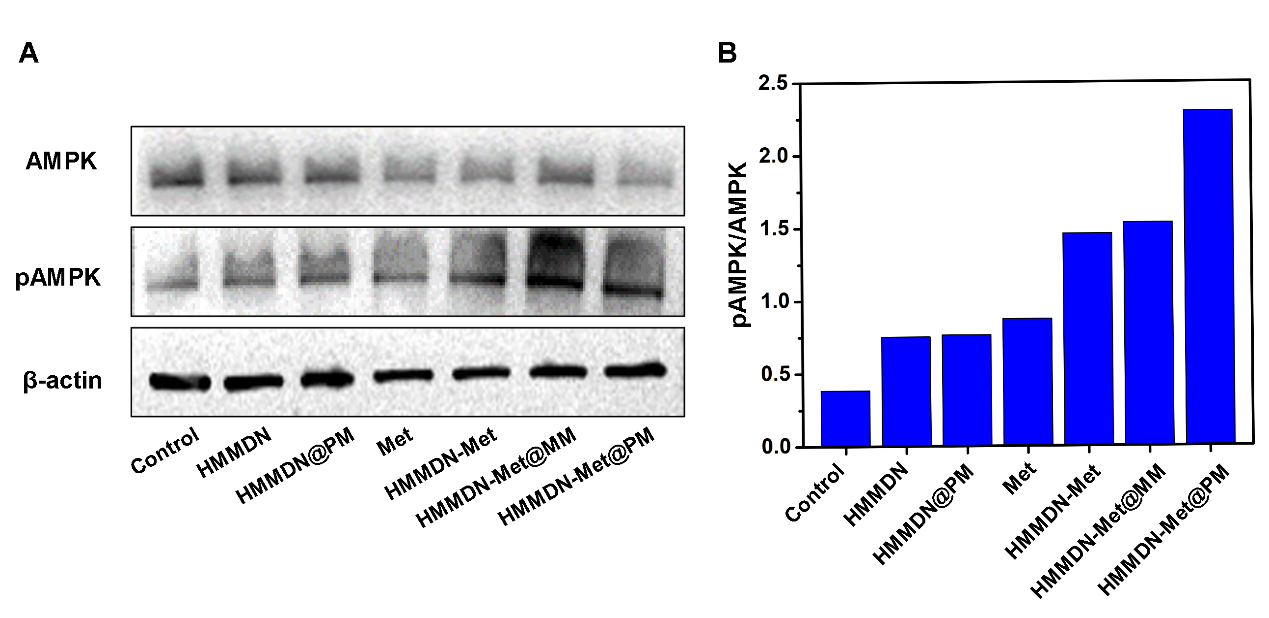


**Figure S4**. **A** Western Blotting analysis of AMPK and pAMPK expression in M2 macrophages with different treatments. **B** Analysis of pAMPK/AMPK in **A**.


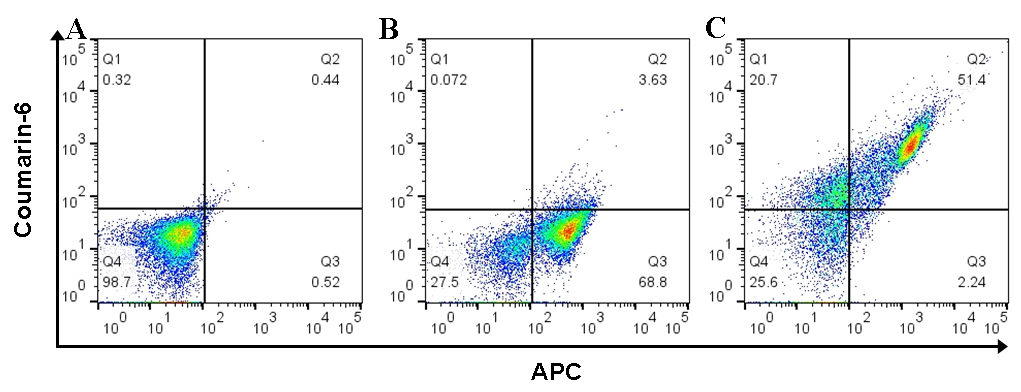


**Figure S5.** Flow cytometric analysis of mixed M0/M1/M2 macrophages **A** left untreated, **B** marked with APC-anti CD206 and **C** treated with HMMDN-C6@PM and marked with APC-anti CD206.

**
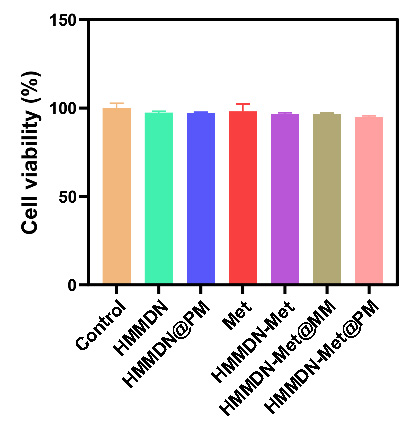
**

**Figure S6.** Cell viability of 4T1 cells incubated with different nanoparticles/Met (Mn, 0.34 mM; Met, 30 μg/mL).
